# Methodological Considerations on the Use of Different Spectral Decomposition Algorithms to Study Hippocampal Rhythms

**DOI:** 10.1523/ENEURO.0142-19.2019

**Published:** 2019-08-01

**Authors:** Y. Zhou, A. Sheremet, Y. Qin, J. P. Kennedy, N. M. DiCola, S. N. Burke, A. P. Maurer

**Affiliations:** 1 Engineering School of Sustainable Infrastructure and Environment, University of Florida, Gainesville, FL 32611; 2 McKnight Brain Institute, Department of Neuroscience, University of Florida, Gainesville, FL 32610; 3 Department of Biomedical Engineering, University of Florida, Gainesville, FL 32611

**Keywords:** EEMD, low γ, nonlinearity, velocity

## Abstract

Local field potential (LFP) oscillations are primarily shaped by the superposition of postsynaptic currents. Hippocampal LFP oscillations in the 25- to 50-Hz range (“slow γ”) are proposed to support memory retrieval independent of other frequencies. However, θ harmonics extend up to 48 Hz, necessitating a study to determine whether these oscillations are fundamentally the same. We compared the spectral analysis methods of wavelet, ensemble empirical-mode decomposition (EEMD), and Fourier transform. EEMD, as previously applied, failed to account for the θ harmonics. Depending on analytical parameters selected, wavelet may convolve over high-order θ harmonics due to the variable time-frequency atoms, creating the appearance of a broad 25- to 50-Hz rhythm. As an illustration of this issue, wavelet and EEMD depicted slow γ in a synthetic dataset that only contained θ and its harmonics. Oscillatory transience cannot explain the difference in approaches as Fourier decomposition identifies ripples triggered to epochs of high-power, 120- to 250-Hz events. When Fourier is applied to high power, 25- to 50-Hz events, only θ harmonics are resolved. This analysis challenges the identification of the slow γ rhythm as a unique fundamental hippocampal oscillation. While there may be instances in which slow γ is present in the rat hippocampus, the analysis presented here shows that unless care is exerted in the application of EEMD and wavelet techniques, the results may be misleading, in this case misrepresenting θ harmonics. Moreover, it is necessary to reconsider the characteristics that define a fundamental hippocampal oscillation as well as theories based on multiple independent γ bands.

## Significance Statement

Fourier, Wavelet and ensemble empirical-mode decomposition (EEMD) converge on conflicting representations of the same time series. Fourier reveals θ harmonics whereas wavelet and EEMD have identified a “slow γ” oscillation. In a comparison of spectral decomposition methods, we find that wavelet and EEMD give the erroneous impression of a slow γ band. Fourier decomposition does not display any spectral deviation that is indicative of slow γ. These data emphasize the importance of multiple analytical approaches with well-understood parameters when decomposing LFP in relation to behavior. On a fundamental level, our analysis points to an imprecise definition of a fundamental rhythm, requiring a reconsideration of both characteristics that define a hippocampal oscillation as well as theories based on multiple γ bands.

## Introduction

Oscillations in the local field potential (LFP) were initially hypothesized to be organized in an energy cascade framework (Bak et al., 1987; Buzsáki and Draguhn, 2004; Buzsáki, 2006). In this framework, higher amplitude, lower frequency rhythms provide the energy into lower amplitude-higher frequency rhythms, suggesting that every frequency is interdependent. Activity across all frequencies reflects a single unified process. This theory had a significant amount of traction, explaining how the cross-scale energy cascade is responsible for the redistribution of power across frequencies and the increase in θ-γ coupling with velocity (Ahmed and Mehta, 2012; Sheremet et al., 2019a,b).

The energy cascade hypothesis, however, was all but abandoned following the report of independent “slow γ” (25–50 Hz) and “high γ” (65–140 Hz) bands (Colgin et al., 2009). The slow γ rhythm identified in the CA1 pyramidal layer correlated with activity in the CA3 region, whereas the higher frequency γ rhythm showed coherence with the medial entorhinal cortex. This led to the two γ hypothesis by which each rhythm is statistically independent and supports dissociable psychological processes (Colgin, 2015b).

The identification of two independent γ rhythms was predicated on a cross-frequency coherence analysis using a Morlet wavelet estimation of power and frequency, showing coherence of slow and fast γ with the θ rhythm (Colgin et al., 2009, their Fig. 1*C*; n.b., 50-Hz alternating current noise overlaps with the θ × slow γ interaction in this figure). Other decomposition methods, however, only found interactions between θ, a single γ (30–80 Hz) and a 120- to 160-Hz oscillation (Zhang et al., 2016), or θ, θ harmonics and a single γ (Sheremet et al., 2019a, their Fig. 6), failing to observe interactions between θ and the slow γ band. Other absences in the detection of slow γ include cross-frequency power correlations or bicoherence analyses (Buzsáki et al., 2003; Masimore et al., 2004; Johnson and Redish, 2007; Sheremet et al., 2016, 2019a). These studies, however, have been eclipsed by reports on slow γ that have reconfirmed the phenomenon through wavelet decomposition (Belluscio et al., 2012; Lasztóczi and Klausberger, 2014, 2016; Tamura et al., 2017; Zhong et al., 2017; Dvorak et al., 2018).

**Figure 1.**
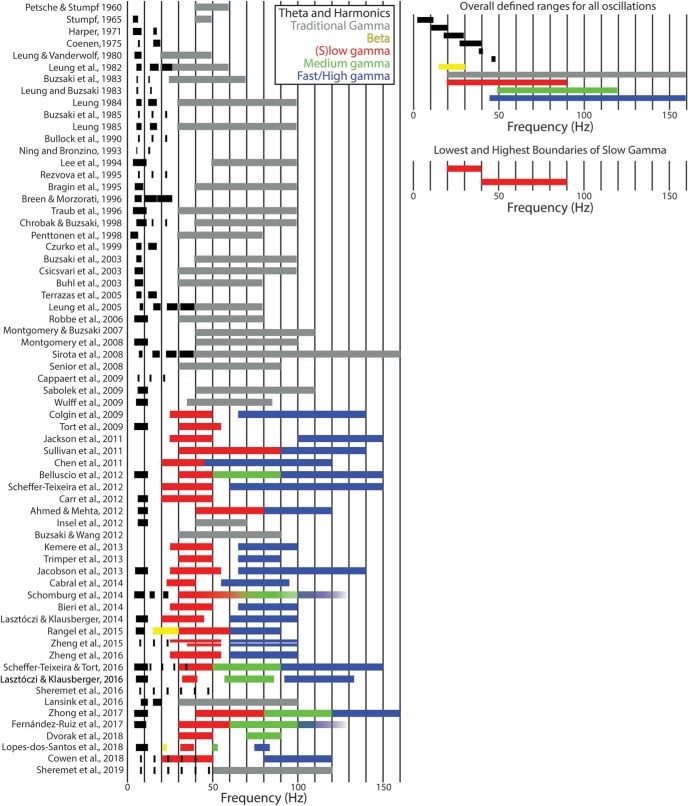
Left, Historical definition of medial temporal lobe oscillatory bands. For each manuscript, a bar spans the range defined for a specific oscillation. In the instance that a range is not defined, but an oscillation is noted (Lopes-Dos-Santos et al., 2018; Sheremet et al., 2019a), a marker covering a short frequency range was used. Furthermore, some ranges were inferred (Sirota et al., 2008 describe a high 32- to 40-Hz harmonic, indicative that the 16- and 24-Hz subdivisions also exist). Note that, before the subdivision of γ into multiple bands, multiple harmonics of θ were reported. For example, Leung et al. (1982) verified the presence of harmonics through bicoherence analysis. Following the observation of multiple γ bands, few manuscripts account for the harmonics of θ. The notable exceptions are Schomburg et al. (2014), cautioning that pyramidal neuron spike modulation in the 20- to 30-Hz band may be related to the third harmonic of θ and not slow γ, and Scheffer-Teixeira and Tort (2016) cautioned that θ wave asymmetry may erroneously contribute to cross-frequency coupling. Cowen et al. (2018) observed up to the fifth harmonic (40+Hz) in both old and young rats which overlapped with their slow γ range, but did not clarify the difference between harmonics and slow γ. Zheng et al. (2015) also increased the lower bound of their slow γ definition in the medial entorhinal cortex in an attempt to avoid the lower order θ harmonics. The recent implementation of bicoherence analysis, however, reveals that the harmonics of θ can extend as high as 48 Hz (Sheremet et al., 2016, 2019a). Note that before the discovery of slow γ, only θ harmonic were reported in the 25- to 50-Hz range. Following the slow γ discovery, reports of harmonics became rare. Right top, An examination of the most extensive defined ranges of all oscillations. Note that θ harmonics spill into the lowest range of traditional and (s)low γ. Therefore, it is evident that harmonics potentially contribute to the γ band up to 50 Hz. No attempt has been made to decipher if the medium γ (Belluscio et al., 2012) was equivalent to fast/high γ across manuscripts. Right bottom, Perhaps more concerning is that when examining the lowest defined ranges (defined by identifying the lowest high pass and the lowest low pass ranges), the (s)low γ band does not overlap across studies, demonstrating inconsistency. Critically, a meaningful definition of what is a fundamental rhythm is missing. Black: θ and θ harmonics; gray: “fast oscillation” and non-subdivided γ; yellow: β; red: (s)low γ; green: medium γ; blue: fast/high γ (Petsche and Stumpf, 1960; Stumpf, 1965; Harper, 1971; Coenen, 1975; Leung and Vanderwolf, 1980; Leung, 1982, 1984, 1985; Buzsáki et al., 1983, 1985, 2003; Leung and Buzsáki, 1983; Bullock et al., 1990; Ning and Bronzino, 1993; Lee et al., 1994; Bragin et al., 1995; Rezvova et al., 1995; Breen and Morzorati, 1996; Traub et al., 1996; Chrobak and Buzsáki, 1998; Penttonen et al., 1998; Czurkó et al., 1999; Buhl et al., 2003; Csicsvari et al., 2003; Leung et al., 2005; Terrazas et al., 2005; Robbe et al., 2006; Montgomery and Buzsáki, 2007; Montgomery et al., 2008; Senior et al., 2008; Sirota et al., 2008; Cappaert et al., 2009; Colgin et al., 2009; Sabolek et al., 2009; Tort et al., 2009; Wulff et al., 2009; Chen et al., 2011; Jackson et al., 2011; Sullivan et al., 2011; Ahmed and Mehta, 2012; Belluscio et al., 2012; Buzsáki and Wang, 2012; Carr et al., 2012; Insel et al., 2012; Scheffer-Teixeira et al., 2012; Jacobson et al., 2013; Kemere et al., 2013; Bieri et al., 2014; Cabral et al., 2014; Lasztóczi and Klausberger, 2014, 2016; Schomburg et al., 2014; Trimper et al., 2014; Rangel et al., 2015; Zheng et al., 2015, 2016; Lansink et al., 2016; Scheffer-Teixeira and Tort, 2016; Sheremet et al., 2016, 2019a; Fernández-Ruiz et al., 2017; Zhong et al., 2017; Cowen et al., 2018; Dvorak et al., 2018; Lopes-Dos-Santos et al., 2018).

To fully appreciate the equivocal foundation of slow γ, it pays to examine the lack of consistency in its described properties across studies. Although harmonics of θ were described relatively early (Harper, 1971) and noted to extend into the 32- to 40-Hz band (Leung et al., 2005), they were not isolated in the initial description of slow γ (Colgin et al., 2009). Furthermore, there is a notable incongruence between the contemporarily defined ranges of slow γ bands across publications (Fig. 1). For instance, the slow γ range defined by Schomburg et al. (2014), 30–80 Hz, broadens the initial range identified by Colgin et al. (2009) and precisely mirrors the filter parameters of Csicsvari et al. (2003), which describes a single γ band. Reverting the filter parameters to ranges used in earlier reports obfuscates the phenomenon being discussed and points to an incongruency in how we define a fundamental hippocampal rhythm. As “fundamental rhythms” are often defined by a substantial “peak above noise” observation (Pesaran et al., 2018; e.g., why spectral whitening is often implemented), it is necessary to understand the initial rationale behind subdividing γ an how this relates to the harmonics of θ, which extend as high as 48 Hz (Sheremet et al., 2016).

The current work revisits prior decomposition techniques employed to identify slow γ frequencies. Our investigation demonstrates that different analysis methods result in contradictory results. Ensemble empirical-mode decomposition (EEMD), as implemented by Lopes-Dos-Santos et al. (2018), fails to account for the higher order harmonics of θ. The analytical parameters chosen in the wavelet analysis of Colgin et al. (2009) produce a corrupt representation of the high-order θ harmonics leading to the appearance of a broad 25- to 50-Hz deflection in the power spectrum. In contrast, θ harmonics are readily identified by short-timescale Fourier decomposition. Critically, spectral decomposition methods as contemporarily implemented yield distinct representations of the same data. To resolve these incongruencies, these three decomposition methods were run on a synthetic benchmark dataset. Notably, both Fourier and wavelet with larger temporal support yielded results closer to the known parameters than EEMD or standard wavelet parameters. The current observations suggest that it is necessary to consider if decomposition methods are appropriately applied and to implement multiple analytical approaches with well-understood parameters when decomposing time-series signals in relation to behavior.

## Materials and Methods

### Subjects and behavioral training

All behavioral and surgical procedures were performed in accordance with the National Institutes of Health guidelines for rodents and protocols approved by the University of Florida Institutional Animal Care and Use Committee. LFP data were obtained from four- to 10-month-old Fisher 344xBrown Norway F1 hybrid rats from the National Institute on Aging colony (Charles River). Datasets used to compare spectral decompositions methods were obtained from three rats (mixed sex cohort: 2 male, 1 female). On arrival, rats acclimated to the colony room for one week. The rats were housed and maintained on a 12/12 h light/dark cycle. All training sessions and electrophysiological recordings took place during the dark phase of the rats’ light/dark cycle. Training consisted of shaping the rats to traverse a circular track for food reward (45 mg, unflavored dustless precision pellets; product #F0021, BioServ). During this time, their body weight was slowly reduced to 85% of their free-feeding baseline. Once the rat reliably performed more than one lap per minute, they were implanted with a custom single shank silicon probe from NeuroNexus or Cambridge NeuroTech. The NeuroNexus probe was designed such that thirty-two recording sites, each with a recording area of 177 µm, were spaced 60 µm apart allowing incremental recording across the hippocampal lamina. The Cambridge NeuroTech probe consisted of sixty-four recording sites, each with an area of 165 µm and spaced 50 µm apart, allowing for 3.15 mm of vertical coverage. In preparation for surgery, the probes were cleaned in a 4% dilution of Contrad detergent (Decon Contrad 70 Liquid Detergent, Fisher Scientific) and then rinsed in distilled water.

### Surgical procedures

Rats were initially sedated in an induction chamber containing 3–5% isoflurane. Once anesthetized, the rat was moved to a nose cone, the head was shaved, and the rat was transferred to the stereotax. During surgical implantation, the rats were maintained under anesthesia with isoflurane administered at doses ranging from 0.5 to 2.5%. The probe implant coordinates targeted the dorsal hippocampus (AP: –3.2 mm, ML: 1.5 relative to bregma, DV: –3.7 mm from brain surface). Once the location of the implant was identified, a 3 × 3-mm contour was drilled out around these coordinates, but not completed. This was followed by the placement of seven anchor screws in the bone as well as a reference over the cerebellum and ground screw placed over the cortex. Once the screws were secured, a thin layer of adhesive cement (C&B-Metabond, Parkell) followed by dental acrylic [Grip Cement Industrial Grade, 675571 (powder) 675572 (solvent); Dentsply Caulk] were applied taking care to not obscure the craniotomy location. Finally, the craniotomy location was completed, irrigating and managing bleeding as necessary once the bone fragment was removed. Next, a portion of the dura was removed, taking care to avoid damaging the vessels and the surface of the neocortex. Small bleeding was managed with saline irrigation and gel foam (sterile absorbable gelatin sponges manufactured by Pharmacia & Upjohn Co). Once the probes were in place, the craniotomy was covered with SILASTIC (Kwik-Sil, World Precision Instruments) and then secured to the anchor screws with dental acrylic. Four copper mesh flaps were placed around the probe providing protection as well as acting as a potential Faraday cage. The wires from the reference and ground screws were soldered to the appropriate pins of the electrode interface board. Adjacent regions of the copper-mesh flaps were soldered together to ensure their electrical continuity, and the ground wire soldered to the copper mesh taking care to isolate the reference from contact with the ground. Once the probe was secured, the rat received 10 cc of sterile saline as well as Metacam (1.0 mg/kg) subcutaneously (the non-steroidal anti-inflammatory is also known as meloxicam; Boehringer Ingelheim Vetmedica, Inc.). The rat was placed in a cage and monitored until fully recovered. Over the next 7 d, the rat was observed to ensure recovery and no behavioral anomalies. Metacam was administered the day following surgery as well. Antibiotics (sulfamethoxazole/trimethoprim oral suspension at 200 mg/40 mg per 5 ml; Aurobindo Pharma USA, Inc.) were administered in the rat mash for an additional 5 d.

### Neurophysiology

Following recovery from surgery, rats were retrained to run unidirectionally on a circle (outer diameter: 115 cm, inner diameter: 88 cm), or figure-8 track (112 cm wide × 91 cm length) for food reward at a single location. The local-field potential was recorded on a Tucker-Davis Neurophysiology System at 24 kHz (PZ2 and RZ2, Tucker-Davis Technologies). The animal’s position was recorded at 30 frames/s (Tucker-Davis Technologies). Spatial resolution was <0.5 cm/pixel. Running speed was calculated as the derivative of the smoothed position. The LFP data were analyzed in MATLAB (MathWorks) using custom-written code as well as code imported from the HOSAtoolbox. Raw LFP records sampled at 24 kHz (Tucker-Davis System) were down-sampled to 2 kHz and divided into fragments of 2048 time samples (∼1 s). To eliminate the effects of anatomic variations in electrode depth between rats, the position of the hippocampal layers with respect to the recording channels was determined by estimating the distribution of current-source density to identify the CA1 pyramidal cell layer (Rappelsberger et al., 1981; Mitzdorf, 1985; Buzsáki et al., 1986; Bragin et al., 1995). Unless otherwise noted, all decomposition analyses were conducted on LFP traces from the CA1 pyramidal cell layer.

### Additional LFP datasets

To investigate cross-frequency interactions and as to not be redundant with previously published data from our lab, LFP recordings from two additional rats from the Buzsáki laboratory were included (https://buzsakilab.nyumc.org/datasets/FernandezRuiz_Oliva/AB1/day11/; https://buzsakilab.nyumc.org/datasets/FernandezRuiz_Oliva/AYA1/AYA1_140808/; sex unknown). The electrode configuration of these probes consisted of eight shanks with 32 sites per shank. Electrode position was determined using current source density analyses (Rappelsberger et al., 1981; Mitzdorf, 1985; Buzsáki et al., 1986; Bragin et al., 1995) triggered to detected ripple events. The distribution of the current sources and sinks of the raw LFP triggered to ripples matched the regional distribution of activity to input layers (Ylinen et al., 1995; Sullivan et al., 2011; Sheremet et al., 2019a). Following current source density analysis, a single channel was selected in the CA1 pyramidal layer of each rat.

### Fourier and wavelet transforms

#### Transforms

The Fourier and wavelet transform can be introduced formally as decomposition on a set of “elementary” functions. In the case of the Fourier transform, the functions form a basis. In the case of the wavelet transform, the most common elementary function sets form frame, a class with weaker properties than a basis. Procedures to extract orthogonal subsets from wavelet frames are available (e.g., the diadic construction; Mallat, 1999). Without going into the details, let *S* be some class of real functions and let *ψ_f_*, with (*f* ∈ *R*), be a basis in *S*; then any *g* ∈ *S* can be written uniquely as

(1) $$G\left(f\right)=\int g\left(t\right)\psi_{f}^{}\left(t\right)dt,g\left(t\right)=\int G\left(f\right)\psi_{f}\left(t\right)df.$$

The “coefficients” *G*(*f*) of the decomposition (also referred to as the transform of *g*) are obtained by taking the inner product of the function *g* with the basis elements [i.e., projecting *g*(*t*) onto the basis], The pair of equations (Eqs. 2, 3) are best known as direct and inverse transforms, sometimes also called analysis and synthesis.

The Fourier transform pair is obtained letting *ψ_f_*(*t*) = *e*2*^πift^*

(2) $$G\left(f\right)=\overset{\infty }{\underset{-\infty }{\int }}g\left(t\right)e^{-2\pi ift}dt,g\left(t\right)=\overset{\infty }{\underset{-\infty }{\int }}G\left(f\right)e^{2\pi ift}df.$$

The wavelet transform and its inverse are given by

(3) $$G\left(s,\tau \right)=\overset{\infty }{\underset{-\infty }{\int }}g\left(t\right)\psi_{s\tau }\left(t\right)dt,g\left(t\right)=\overset{\infty }{\underset{-\infty }{\int \int }}G\left(s,\tau \right)\psi_{s\tau }\left(t\right)dsd\tau ,$$

where the functions $\psi_{s,\tau }\left(t\right)=\alpha \varphi \left(\frac{t-\tau }{s}\right)$ are “copies” of the “mother wavelet” *φ*(*t*), shifted in time by *τ* and scaled by *s*, with *α* a normalization constant. The full set of wavelets {*ψ_s_*_,_*_τ_*(*t*)}*_s_*_,_*_τ_*_∈_*_R_* is in general complete but not independent (i.e., larger than a basis). The wavelet representation was designed for two main applications: compression and resolving short-time transient coherent structures such as solitons and chirps. For these purposes, the lack of orthogonality of the decomposing modes is not an inconvenience, because the structure of the decomposition in the dual space is not important. Compression algorithms only need a small number of modes and the synthesis rule for reconstructing the signal, while the identification of transients focuses on optimizing the time-scale localization (see the discussion of the time-scale atoms below). The lack of orthogonality of the wavelet elementary function set creates problems in applications where one seeks to assign meaning to the actual components of the decomposition. Here, meaning means repetition (an implied stochastic process model) and requires the ability to attribute variance unequivocally to each of the decomposition modes. For such an application, the lack of orthogonality of the wavelet decomposition becomes a major inconvenience, because one cannot attribute unique variance to any single wavelet mode.

#### Discrete transforms

Unless the set of functions are carefully defined, Equations 2, 3 are only formal, and in most cases, they do not have any elementary mathematical meaning; when they do, their usefulness is limited. For example, if *g*(*t*) = 1, the Riemann integral in the equation does not exist; however, a rather restrictive elementary theory can be built for *T*-periodic functions.

Evolving the generic Fourier and wavelet equations into useful analysis tools has complexities that required the development of full theories (e.g., the theory of distributions, or generalized functions; Schwartz, 1950; Lighthill, 1958). For example, in Fourier equations *ψ_f_*(*t*) = *e* ^2^*^πift^* are orthogonal in the sense that $\overset{\infty }{\underset{-\infty }{\int }}e^{2\pi ift}dt=\delta \left(f\right),$ where *δ* is the Dirac delta function (Vladimirov, 2002; Strichartz, 2003). A complete discussion of these is far beyond the scope of this study, and is also unnecessary, because time series measured in practical applications are always of finite length *T*, sampled at time intervals Δ*t* (*T* = *n*Δ*t*), that is., are finite sequences of real numbers *g*(*t*) = {*g*_1_, *g*_2_, ⋯, *g_N_*}, with *g_j_* = *g*(*t_j_*). Such sequences naturally form *N*-dimensional spaces, in which integral Transforms, such as the Fourier or wavelet equations, are represented by finite-dimensional linear operators, that is, *N* × *N* matrices. These discretized versions of the Fourier and wavelet representations are called the discrete transforms (Briggs, 1995; Mallat, 1999; Strang, 2006).

The discrete Fourier transform pair is (in MATLAB convention)

(4) $$G_{m}=\frac{1}{N}\overset{N-1}{\underset{n=0}{\sum }}g_{n}\psi_{mn},g_{n}=\overset{N-1}{\underset{m=0}{\sum }}G_{m}\psi_{mn}^{*},\psi_{mn}=e^{-2\pi i\frac{mn}{N}},$$

where $\psi_{mn}=\psi_{m}\left(t_{n}\right)=e^{-2\pi if_{m}t_{n}}$ are the basis vectors. This pair of equationsare sometimes called the analysis and synthesis of the signal *g*(*t*).

Here, *f_m_* = *m*Δ*f* and *t_n_* = *n*Δ*t* represent the discretized frequency and time grids, with $\text{Δ}f=\frac{1}{T},$ and $\text{Δ}t=\frac{T}{N}$. Basis functions are orthogonal in the sense that, for any integer*m*, with *m* ≠ 0 and *m* ≠ *N*, $\overset{N-1}{\underset{n=0}{\sum }}e^{-2\pi i\frac{mn}{N}}=0$.

A discrete version of wavelet transform is

(5) $$G_{mn}=\overset{N-1}{\underset{k=0}{\sum }}g_{k}\varphi_{mnk},\text{ with }\varphi_{mnk}=s^{-\frac{m}{2}}\varphi \left[s^{-m}\left(k-n\text{Δ}\tau s^{m}\right)\right]$$

where Δ*τ* is a time-shift increment. The wavelets *φ_mnk_* form orthogonal only for compact-support wavelet shapes *φ* (e.g., Haar, and Daubechies wavelets; Daubechies, 1988, 1992; Mallat, 1999).

The Parseval relation ensures that the discrete Fourier equations conserve the variance of the time sequence and its transform (Briggs, 1995), i.e., *σ^g^* = *σ^G^*, where *σ^g^* = Σ*_n_*|*g_n_*|^2^ is the variance of *g*. The discrete wavelet transform does conserve variance (*σ^g^* ≠ *σ^G^*) and in general, the variance ratio depends on *g*, which means that a universal correction factor does not exist.

#### Windowed Fourier transform (WFT)

To investigate the evolution of spectra over time, WFT is introduced where the original signal is multiplied by a window function which is nonzero for only a short period of time

(6) $$G^{win}\left(\tau ,f\right)=\overset{\infty }{\underset{-\infty }{\int }}g\left(t\right)w\left(t-\tau \right)e^{-2\pi ift}dt$$

where *w* is the window function (Priestley, 1981; Roads, 2004). The window function slides along the time axis, which gives rise to a time-frequency representation of the original time series. This time-frequency representation can be plotted as the spectrogram. Typically, window functions are smooth, “bell-shaped,” time-localized curves, the Fourier transform of the window function is

(7) $$W\left(f\right)=\overset{\infty }{\underset{-\infty }{\int }}w\left(t\right)e^{-2\pi ift}dt.$$

In frequency space, the window function *w* is usually composed of a bell-shaped main lobe and symmetric side lobes. The bandwidth of a window function is defined as

(8) $$B=\frac{\int_{-\infty }^{\infty }{\left|W\left(f\right)\right|}^{2}df}{{\left|W\left(f\right)\right|}_{max}^{2}}.$$

**Figure 2.**
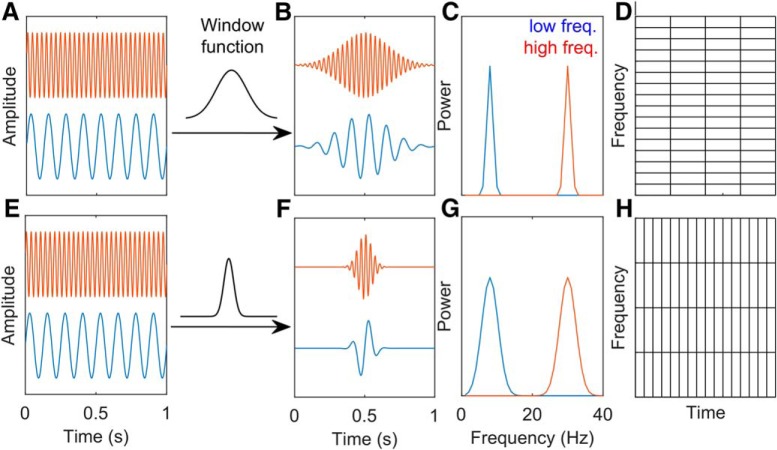
WFT atoms. ***A***, For WFT, a window function is applied to all the frequency components. ***B***, Adoption of a Hanning window with wide time support to fast and slow oscillations, which (***C***) gives rise to fast and slow oscillations that decay slowly in the time domain. ***D***, In the frequency domain, the power (amplitude square) of these slowly decaying oscillations have a relatively narrow frequency band, and the bandwidth is the same for fast and slow oscillations. This results in a transform with coarse time resolution but fine frequency resolution. ***E***, On the contrary, applying a Hanning window with narrow time leads to (***F***) fast decay oscillations in the time domain. ***G***, However, these fast decay oscillations have a wide frequency band, which (***H***) results in a transform with fine time resolution but coarse frequency resolution.

The spectrum estimation depends critically on the window bandwidth as it will smooth the spectrum and influence the frequency resolution (Fig. 2). The time-frequency resolution of transforms will be further discussed in the following section.

#### EEMD

**Figure 3.**
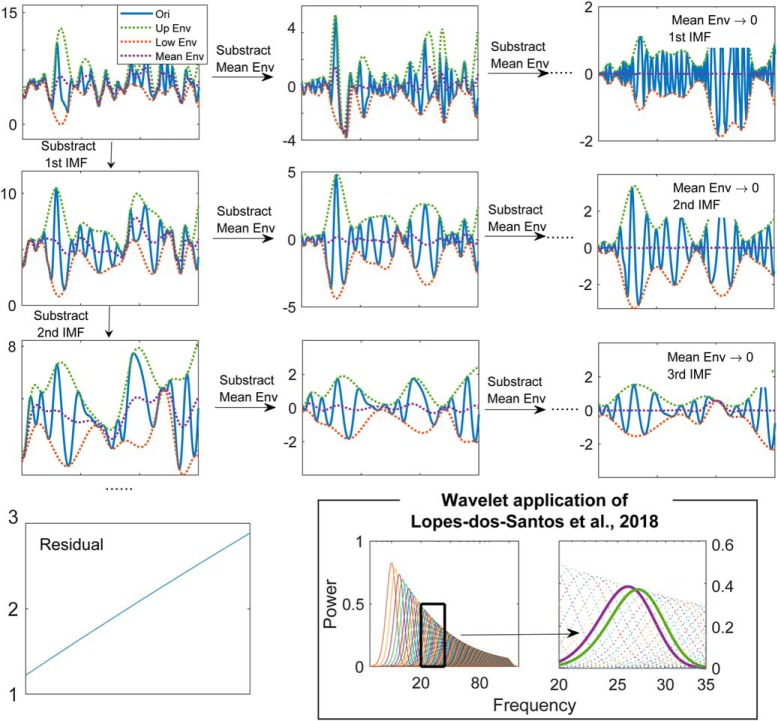
Description of empirical mode decomposition and the wavelet analysis as used by Lopes-Dos-Santos et al. (2018). For a given time series, the mean envelope can be obtained by averaging upper envelope and lower envelope. Subtracting the mean envelope from the original time series and repeating this process until the time series has an almost symmetric upper and lower envelopes. The obtained time series is defined as the first order intrinsic mode function (the 1st row in the figure). Subtracting the 1st intrinsic mode function from the time series and repeating the process, we can get high order intrinsic mode functions (the 2nd and the 3rd rows). Following this process, the time series will be decomposed into intrinsic mode functions and a residual, which has overlapped upper and lower envelopes. Inset, Rather than use the wavelet steps of Colgin et al. (2009), Lopes-Dos-Santos et al. (2018) opted to use steps of 1 Hz between Morlet wavelets making for a highly redundant, overly convolved representation.

A brief description of empirical mode decomposition (EMD) is given in Figure 3, where the original time series is decomposed into intrinsic mode functions and a non-oscillatory residual. The intrinsic mode functions generated by EMD have two properties: (1) the number of extrema and the number of zero-crossings will either be equal or differ at most by one; (2) the upper envelope and lower envelope are symmetric. These two properties guarantee the intrinsic mode functions have a well-behaved Hilbert transform. However, the decomposing process doesn't ensure orthogonality of intrinsic mode functions, and except for those two properties, we have limited prior knowledge about the decompose results. The question, why the symmetric mode functions are favorable, and why a well-behaved Hilbert transform is desired should be asked before applying the EMD. However, EMD suffers from the mode mixing problem which renders the algorithm unstable (Gledhill, 2003). To solve this issue, EEMD is proposed. In the EEMD algorithm, white noise is added to the original signal before decomposition to alleviate mode mixing and is canceled out after the decomposition by averaging over ensemble (Wu and Huang, 2009). In this study, following the procedure of Lopes-Dos-Santos et al. (2018), the ratio between the variance of added white noise and the original LFP was 0.5, and the ensemble number was 200. After applying the EEMD, the power spectrum for each intrinsic mode function was used to identify the peak frequency. The supra-θ signal is defined as the sum of all the intrinsic mode functions with peak frequency above 12 Hz. The time-scale power distribution of supra-θ signal was obtained by Morlet wavelet transform with a constant ratio for the wavelet family set as 7 (Colgin et al., 2009; the meaning of this parameter is introduced in the numerical implementation section).

#### Time-frequency atoms

For the Fourier transform, the duration *T* of the time sequence and the frequency resolution Δ*f* of the transformed sequence are related through the reciprocity relation *T*Δ*f* = 1, which implies that increasing the frequency resolution Δ*f* is equivalent to increasing the time duration *T* of the analyzed signal. The *T*Δ*f* = 1 equation highlights an important limitation of the Fourier transform: if *g*(*t*) is highly localized, its transform *G*(*t*) has a wide frequency support. This means that very high sampling rates cover the wide frequency domain, and the interpretation of the high-frequency content can become difficult. Restricting the Fourier analysis equation to a specified duration *T* is equivalent to multiplying the time series *g*(*t*) by finite support rectangular window *w*(*t* – *τ*) = 1 if |t−τ|≤T2 and zero otherwise. In other words, the integral operator

(9) ∫τ−T2τ+T2dte−2πift

may be written as

(10) $$\overset{\infty }{\underset{-\infty }{\int }}dt\text{\&}w\left(t-\tau \right)e^{-2\pi ift}=\overset{\infty }{\underset{-\infty }{\int }}dt\varphi_{f\tau }^{*}\left(t\right),$$

which may be interpreted as a projection of *g* onto functions *φ*(*t*) = *w*(*t*)*ψ*(*t*). The function *φ* could be described as a localized oscillation. If one constructs in the time-frequency plane a rectangle of sides *T* and Δ*f* centered, say, at $t_{0}=\frac{T}{2}$ and $f_{0}=\frac{T\text{Δ}f}{2\text{Δ}t}$, the *T*Δ*f* = 1 equation states that the area of this rectangle is constant, regardless of the value of *T*. This rectangle is sometimes called the Heisenberg box (Mallat, 1999). This is in fact an example of the application of the general Heisenberg uncertainty principle, which states that the area of a Heisenberg box cannot be made arbitrarily small. For an arbitrarily-shaped localized oscillation *φ*(*t*) with Fourier transform *φ*(*f*) and unit variance $\left(\overset{\infty }{\underset{-\infty }{\int }}{\left|\varphi \right|}^{2}dt=\overset{\infty }{\underset{-\infty }{\int }}{\left|\phi \right|}^{2}dt=1\right)$, defining the time and frequency widths as

(11) $$\sigma_{t}=\overset{\infty }{\underset{-\infty }{\int }}{\left(t-t_{0}\right)}^{2}{\left|\varphi \right|}^{2}dt,\;\sigma_{f}=\overset{\infty }{\underset{-\infty }{\int }}{\left(f-f_{0}\right)}^{2}{\left|\phi \right|}^{2}dt,$$

one can show (Gabor, 1946; Percival and Walden, 1993; Mallat, 1999) that $\sigma_{t}\sigma_{f}\geq \frac{1}{4\pi }$.

**Figure 4.**
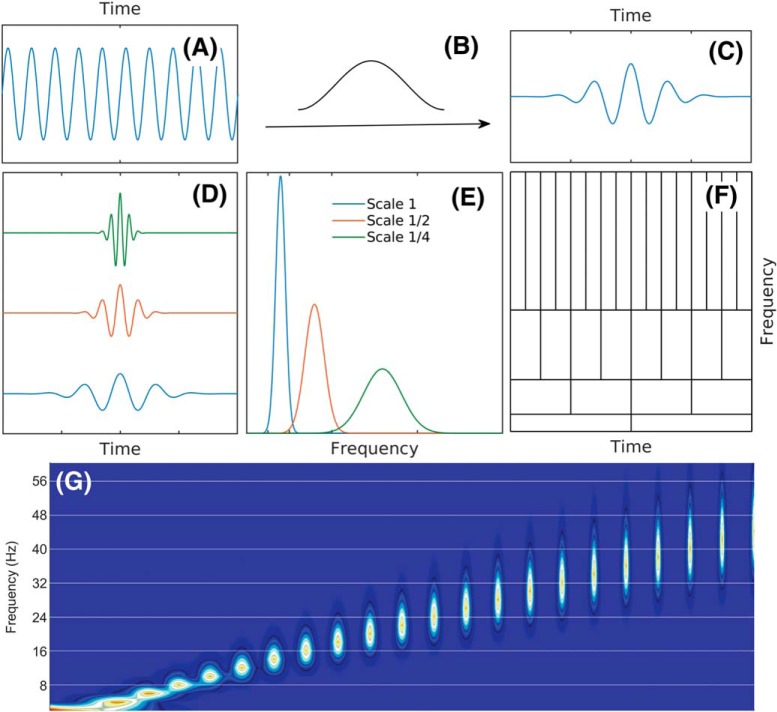
A schematic of the Heisenberg boxes of the wavelet transform. ***A–C***, The mother wavelet is obtained by the multiplication of a sine function with a window. ***D***, Wavelets at three scales resulting by the scaling of the mother wavelet. ***E***, Frequency distribution of the power of wavelets. ***F***, Heisenberg boxes of the wavelet transform. ***G***, Frequency distributions of the first 22 wavelet scales used in Colgin et al. (2009). Note that the 8-Hz bin only overlaps with approximately three wavelets, 16 Hz overlap with nearly five wavelets, 24 Hz overlaps with approximately seven wavelets, and 32 Hz overlaps with more than 10 wavelets. It is this decimation that results in distributing the power of the 24-Hz and higher harmonics of θ over multiple frequency bands. The implementation of Lopes-Dos-Santos (2018) accentuates this effect through their use of 1-Hz steps between Morlet wavelets.

In other words, it is impossible to achieve simultaneous arbitrary resolutions both in time and frequency. While the time-frequency resolution (area of Heisenberg boxes) cannot be made arbitrarily small, it can be minimized. Gabor (1946) showed that the minimal area for a Heisenberg box is achieved by localized oscillation *φ*(*t*) = *w*(*t*)*e* ^2^*^πift^* where *w* is a Gaussian-shaped window. Following his work, Goupillaud et al. (1984) used this shape, later called the Morlet wavelet, to introduce the continuous wavelet transform (Grossmann and Morlet, 1984; Mallat, 1999). The limitations imposed by the Heisenberg uncertainty principle are illustrated in Figure 4. A Morlet wavelet results as a product of a sine function with a Gaussian window. The resulting function is the mother wavelet. The wavelet transform then uses scaled versions of the mother wavelet as elementary functions. It is important to note that the wavelets shown in Figure 4*D*, are not harmonic; therefore, they are not uniquely characterized by a single frequency value; instead, they are characterized by a frequency interval, say, corresponding to the width of the frequency spread of their power (as given by the Fourier transform). The frequency distribution of power is, as expected, narrower for the larger-scale wavelets, and wider for smaller-scale wavelets. Therefore, the wavelet Heisenberg boxes coverage of the frequency axis is not uniform, and consequently, using wavelets as a “frequency” representation results in a non-uniform frequency resolution, with resolution degrading at higher frequencies.

It is worth noting that, theoretically, wavelet transform can acquire arbitrary frequency resolution at a given frequency range by dilating the width of the mother wavelet. However, an appropriate choice of mother wavelet requires a good understanding of the process under investigation. In the analysis of θ harmonics, if we want to obtain the frequency resolution of 2 Hz when the central frequency is around 30 Hz, the mother wavelet will have ∼20 oscillations before decaying (compare Fig. 4*D*, which is the typical wavelet used in the confirmation of slow γ analysis). Thus, it is worth noting that wavelet is capable of resolving harmonics, but not in the implementation of Colgin et al. (2009).

#### Numerical implementation

Fourier and wavelet analysis procedures were implemented in the MATLAB environment using available MATLAB toolboxes. The Fourier spectrum was estimated by dividing the LFP time series into 2048-point (1 s) segments sorted by rat speed and windowed using a Hamming window. The wavelet transform used the Morlet wavelet, with the central frequency of the Morlet wavelet defined as the nominal frequency. The Morlet wavelet family is characterized by the constant ratio between the central frequency of wavelet and SD of the applied Gaussian window, which is set to 7 in our analysis to match the methods of Colgin et al. (2009).

The wavelet scalogram is defined as the logarithm of the squared modulus |G_mn_|^2^ of the wavelet coefficients. The power spectral density (PSD) for the wavelet transform was computed as the time marginal of the wavelet transform (Abry et al., 1993). The Fourier spectra were estimated using the Welch method (Welch, 1967).

#### Methodological limitation

At this point, it needs to be stressed that the wavelet and EEMD assignment of variance to a specific frequency (e.g., in the construction of a PSD) is ultimately arbitrary and potentially meaningless since the elementary functions used for the decompositions are not harmonics functions (sine/cosine). The data presented here are for illustrative and replication purposes. We believe this methodological treatment is flawed and should be treated with circumspection.

### Bicoherence analysis

Higher-order spectra provide information regarding the degree of cross-frequency coupling. The bispectrum, the “lowest” of the high-order spectra, has had a long history in the field of wave dynamics (Hasselmann et al., 1963; Rosenblatt and Van Ness, 1965; Coppi et al., 1969). The bispectrum has been thoroughly reviewed in terms of both statistical and mathematical background (Harris, 1967) as well as its application to nonlinear wave interaction (Kim and Powers, 1979). Furthermore, the relationship between a time series and the third-order statistic is well understood (Haubrich and MacKenzie, 1965; Masuda and Kuo, 1981; Elgar, 1987). As noted previously (Sheremet et al., 2016; Kovach et al., 2018), bispectral analysis (the Fourier transform of the third-order cumulant) quantifies the degree of phase-envelope amplitude coupling between the frequencies of the LFP, while the bicoherence quantifies the degree of cross-frequency coupling independent from the amplitude (Barnett et al., 1971; Ning and Bronzino, 1989; Sigl and Chamoun, 1994; Bullock et al., 1997; Muthuswamy et al., 1999; Hagihira et al., 2001; Gloveli et al., 2005; Li et al., 2009; Wang et al., 2017; Shahbazi Avarvand et al., 2018). It is emphasized here as well as elsewhere (Van Milligen et al., 1995; Pradhan et al., 2012) that the bispectrum measures phase coupling, defined to occur when the sum of phases between two frequencies is equal to the value of a third frequency plus a constant. Furthermore, the real and imaginary part of the normalized bispectrum provides information regarding how cross-frequency interactions contribute to skewness and asymmetry, respectively (for more information, see Sheremet et al., 2016, 2019a). Although it is starting to become common practice to account for asymmetry, a cnoidal wave is both symmetric and nonlinear which will cast harmonics when decomposed. Therefore, bicoherence analysis assures that all forms of distortion that result in harmonics can be accounted for (Aru et al., 2015). Notably, traditional measures of phase-amplitude coupling tend to fall short when compared to bispectral analysis (Kovach et al., 2018). For a Fourier based approach that assesses hippocampal phase amplitude coupling, we refer to our prior publication (Sheremet et al., 2019a). A thorough discussion of bicoherence and bispectral analysis, are beyond the scope of the current paper, and have been described in detail elsewhere (Dumermuth et al., 1971; Aru et al., 2015; Kovach et al., 2018).

### Power-power correlation

As the hippocampal LFP transitions between quiescent activity with intermittent sharp-waves/ripples during rest and high-amplitude θ during awake-behavior, the time series at long time scales is undeniably non-stationary (that is, the mean and variance of the recorded voltage change over time). Therefore, the use of an average power spectrum over large time series has a marginalized meaning as it can average out intermittent events. To address this, a simple way to determine how the power spectra changes over time and investigate frequency interactions is to calculate the correlation coefficients across different Fourier transforms (Masimore et al., 2004, 2005) using locally stationary segments of LFP. For instance, should an increase in the 8-Hz rhythm be accompanied by a decrease in the 12-Hz range, a negative correlation should exist. Furthermore, if the spectrum undergoes local change (e.g., increases in the 8-Hz rhythm is accompanied by an increase in the adjacent 7- and 9-Hz band, a non-zero width will be present (Masimore et al., 2004, 2005). Velocity is a primary driver of variance for both θ and γ (Sheremet et al., 2019a,b). Thus, all velocities were considered together, as this approach enabled the parametric space to detect potential relationships across frequency bands.

### Power triggered spectral decomposition

Stationarity describes the property of a time series in which the overall characteristics, such as the average and variance of the power, remain constant over time. It has been previously argued that slow and fast γ rhythms are non-stationary and highly transient. However, nonstationary events, such as ripples, can be treated as “locally stationary” should it be possible to detect the event and in turn, realize the spectra (Pesaran et al., 2018). This approach allows one to discuss statistical characteristics of ripples such as the average frequency and power. Knowing that slow γ is defined by a frequency range between 25 and 55 Hz, and these theoretical events have been detected by instances of power that exceed 2 SDs above the mean power (Bieri et al., 2014), then it should be possible to calculate the Fourier spectra on slow γ using this approach. The utility of detecting such transient events can be compared by running the same procedure on ripples.

Therefore, instances of high-power slow γ events or ripple events were identified as described previously (Maurer et al., 2006; Skaggs et al., 2007). Briefly, the raw LFP was filtered either in 25- to 55-Hz range (slow γ) or 120- to 250-Hz range (ripple) and rectified by squaring the filtered trace. The mean and SD of the upper envelope of the rectified trace was calculated. For every instance in which the envelope exceeded the mean plus 2 SDs of the entire trace, 2 s of the raw LFP was extracted and spectrally decomposed using either Fourier decomposition with Thomson’s multi-taper or wavelet decomposition.

### Spike frequency analysis

Often the presence or absence of an oscillation is buttressed by whether single-unit firing is modulated at a specific frequency by examining the phase relationship between an oscillation and neuron spiking. Thus, to determine the frequency in which neuronal spiking occurred, we implemented spectral analyses on spike trains (Leung and Buzsáki, 1983; Sheremet et al., 2016). Single-unit data were generously provided by the Buzsáki laboratory and curated by the Collaborative Research in Computational Neuroscience (Mizuseki et al., 2014; Pastalkova et al., 2015; seven datasets: maze05.005, maze06.002, i01_maze06.005, i01_maze08.001, i01_maze08.004, maze13.003, and maze15.002). For these datasets, the rat performed a delayed alternation task on a figure-8 maze, running in a wheel during the delay. Only datasets in which CA1 neuron recordings were obtained bilaterally were used for the analysis. Action potentials for pyramidal cells and interneurons were initially sorted into velocity bins, analyzing the 5–15 or 35+ cm/s conditions separately. These spike times were converted into a binary time series with a sampling frequency of 1250 Hz. As this bin size is slightly smaller than the traditional window of 1 ms used for waveforms, a boxcar convolution was performed such that a single spike registers as one across three adjacent bins. This output was then passed through the pmtmPH (https://www.mathworks.com/matlabcentral/fileexchange/2927-pmtmph-m) function to calculate the PSD based on multi-taper analysis. Each unit’s power was normalized by the total power and then sorted by peak θ frequencies to align the harmonics between units. The average and standard error power spectra are presented.

### Spike-LFP coherence

The general theory behind spike-LFP coherence is that synaptic events, which are the primary component in shaping the local-field potentials (Buzsáki et al., 2012), are responsible for generating action potentials, which in turn generate further synaptic events (Pesaran et al., 2018). Thus, if action potentials occur in support of a fundamental rhythm, then a peak in spike-field coherence may be evident even in the absence of a peak in the LFP spectrum (Pesaran et al., 2018). Therefore, using the CRCNS datasets from the Buzsáki laboratory described above, we calculated the spike-LFP coherence (Leung and Buzsáki, 1983) as a function of running speed.

### Spike preferred frequency of modulation analysis

While spike-LFP coherence quantifies the degree to which neurons fire to a particular phase of an oscillation, in related studies, spike-LFP coupling was determined through a modulation analysis (Colgin et al., 2009; Schomburg et al., 2014). Therefore, we revisited these analyses. Using the CRCNS data, the phase of neurons was calculated relative to a narrow band filter of the LFP, in 1 Hz steps from 2–120 Hz. Cells were considered to be phase locked if they differed significantly from a uniform distribution (using a *p* < 0.01 as in Schomburg et al., 2014). In the instance that the cell exhibited significant phase locking, the preferred phase, and depth-of-modulation (Skaggs et al., 1996) was calculated. For all significant cells, a mean modulation index providing the phase and vector magnitude of modulation can be realized for each frequency. Finally, this approach provides the ability to determine the fraction of cells that are significantly modulated at each frequency as well as the primary preferred frequency of each neuron. To be included in this analysis, neurons must have had an average firing rate above 0.25 Hz.

## Results

### Investigation of cross-frequency interactions within the hippocampus

We have previously conducted an extensive investigation of cross-frequency interactions between θ and γ in the hippocampus (Sheremet et al., 2019a). Using filterless approaches of power-power correlations (Masimore et al., 2004, 2005) and phase-coupling assessed through bicoherence analysis (Sheremet et al., 2016, 2019a), we found interactions between θ, harmonics, and a broad unitary 50- to 120-Hz band. Notably absent in our results was the 25- to 55-Hz slow γ band. Instead, at high velocities, frequencies between 25 and 55 Hz were dominated by the harmonics of θ. While it could be argued that slow γ is transient to the point of being averaged out (considered below), the same transience argument has been made for the 60- to 100-/140-Hz fast γ rhythm (Colgin et al., 2009; Bieri et al., 2014), which was resolved by both cross-frequency analytical approaches. With this consideration that there may be something unique to our rats or data collection methodologies that preclude finding slow γ, and to enhance scientific rigor we revisited cross-frequency interactions using data from two rats generously made available from the Buzsáki laboratory (see Materials and Methods).

**Figure 5.**
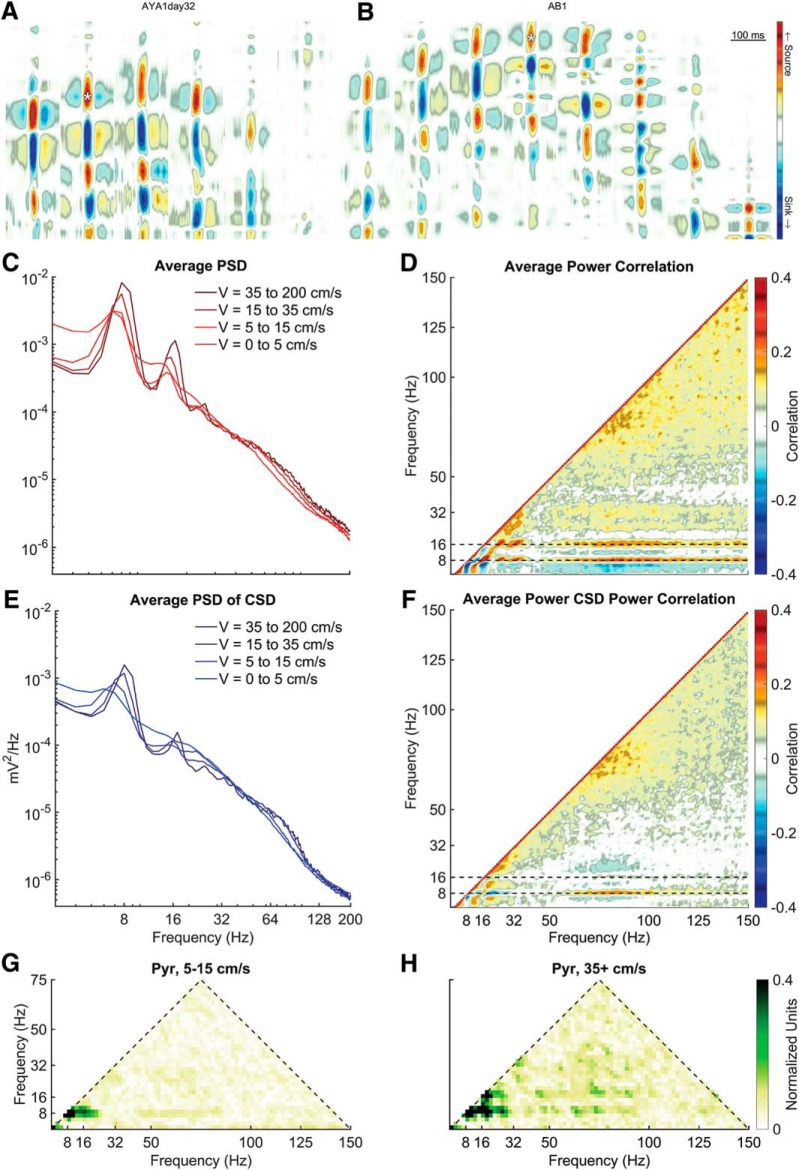
Cross-frequency interactions as a function of velocity. ***A***, ***B***, Current source density plots of local-field potential data triggered to ripples for two rats generously made available by the Buzsáki laboratory. Using the source-sink profile, layers can be easily identified. The location of the white asterisks depicts the channel location selected for each rat for the following analyses. ***C***, Rat average CA1 pyramidal layer PSD as a function of velocity. Overall, the PSD has an inverse relationship between amplitude and frequency (A = f^-α^) such that lower frequencies are higher power. As running speed increases, the power of θ as well as the peaks at 18 and 27 Hz increase with velocity. There is also an associated increase in the 50- to 120-Hz range with velocity (described as a moving front; Sheremet et al., 2019b). At low frequency and within the inter-harmonic intervals, there is a loss of power. ***D***, To examine the redistribution of power across frequencies, multiple different PSD realizations were cross-correlated. Major regions of interactions include what appears to between harmonics of θ and between θ and a broad γ band >50 Hz. Negative correlations are evident between frequencies that lose power with running speed (e.g., <6 Hz) and those that increase in power with running speed (e.g., 9 Hz). ***E***, In the instance that slow γ may be overpowered by a volume conducted oscillation, the PSD was also conducted on the same CA1 pyramidal channel after calculating the current source density using the adjacent channels. The redistribution of power becomes more evident in this depiction with the inter-harmonic intervals losing power as the θ and harmonics increase in power. ***F***, The PSD correlation of the current source density trace from the CA1 layer. Note little interaction between 32-50 Hz and θ, suggesting that this band range does not demonstrably change as a function of velocity. ***G***, ***H***, Average bicoherence of the CA1 LFP at low and high velocities, respectively. Note that while there is a three-wave interaction among the harmonics of θ (Sheremet et al., 2016), there is a notable dearth of interaction in the slow γ range.

Using current-source density, an “electroanatomic” reconstruction of position can be conducted to identify recording location (Berényi et al., 2014). As the CA1 pyramidal layer was the site of the initial description of slow γ (Colgin et al., 2009) as well as recent replication (Lopes-Dos-Santos et al., 2018), the electrode in the layer was identified based on current source densities triggered to ripple events (Fig. 5*A*,*B*). In agreement with our prior publications (Sheremet et al., 2016, 2019a,b), there are multiple prominent peaks in the CA1 pyramidal layer that align with 7–10, 14–20, and 21–30 Hz indicative of θ harmonics (Fig. 5*C*). Within the 50- to 120-Hz range, there was a mild increase in power with velocity (Ahmed and Mehta, 2012). While θ and the harmonics increased, the frequency bands less than θ, between θ and the first harmonic and between the 18- and 27-Hz harmonic lost power with velocity. Finally, there was a redistribution of power between the 27-Hz harmonic and the start of the 50- to 120-Hz γ range.

The power spectra were calculated for non-overlapping 1-s segments. By correlating the vector of power of one frequency band with another, it is possible to determine whether there is a positive or negative relationship (Masimore et al., 2004, 2005). As can be seen in Figure 5*D*, as θ power increases, there is a concurrent increase in θ harmonic power, as indicated by the multiple low-frequency correlations. Furthermore, there is a correlation between θ, the first harmonic (18 Hz) and a broad, unitary γ (50–130 Hz). Note that there is an inverse correlation between the frequencies <9 Hz and the unitary γ band, revealing that the 50–130 Hz increases in power while the low frequencies lose power. That is, while θ and the harmonics increased in power, the inter-harmonic intervals lost power. This redistribution of power is reflected in the positive correlation between the sub θ frequency and the ∼14- to 15-Hz band. Finally, there is little in the way of structure between the 36 Hz θ harmonic and the start of the unitary γ band at 50 Hz.

While this is descriptive of the LFP, it does not account for the potential effects of volume conduction. Therefore, to ensure that our observations were carried by local voltage changes, the PSD of the current source density was calculated for the pyramidal layer. Specifically, any signal that was common to the electrode above and below the pyramidal cell electrode was removed. Once again, as velocity increases, there was a redistribution of power such that θ and harmonics become more prevalent. Power also increased in the 50- to 120-Hz range. Moreover, there was a loss of power in the inter-harmonic intervals (Fig. 5*F*). The average power correlation of the current source density reflects this observation with positive correlations between θ and its harmonics, θ, and the 50- to 130-Hz range and the inter-harmonic intervals. Negative correlations were notable between θ and the inter-harmonic intervals and γ and the inter-harmonic intervals. Again, there was little structure between the last interaction indicative of a harmonics and the start of the 50- to 130-Hz interaction, which is the range typically corresponding to slow γ.

Therefore, the data up to this point do not support the idea there is a slow γ band that interacts with θ. θ Has long been documented to increase in power with velocity (Whishaw and Vanderwolf, 1973; Morris and Hagan, 1983; Rivas et al., 1996; Shen et al., 1997; Maurer et al., 2005). Should there be any systematic change in slow γ with velocity, it should be evident in the power correlations (Fig. 5*D*,*F*). However, as θ power increases, the slow γ band neither increase nor decreases. It is worth noting here that the literature is obtuse regarding changes in slow γ with velocity: Zheng et al. (2015) reported a minimal change with increasing velocity, Kemere et al. (2013) reported a decrease, Chen et al. (2011) found an increase, and Ahmed and Mehta (2012) suggest that there is only one γ band that increases in frequency and power with velocity. In light of this ambiguity, any potential result risks being construed as supporting slow γ. For instance, the power of slow γ is not modulated by the power of θ, but the interaction resides in phase coupling. Or it may be argued that the highest correlation observed near 36 Hz is actually slow γ.

Therefore, to address these two possibilities, we implemented bicoherence analysis to quantify the degree of cross-frequency phase coupling in the LFP (see Materials and Methods). Bicoherence is uniquely suited to identify harmonics as well as other forms of phase coupling (Aru et al., 2015; Sheremet et al., 2016). As slow γ phase has been reported to be coupled to θ phase (Belluscio et al., 2012; Colgin, 2015a), should this be true, then an interaction should be evident.

In congruence with our prior reports, the presence of θ harmonics became more prominent with running speed across all layers (Sheremet et al., 2016, 2019a; Fig. 5*G*,*H*). There is a peak at 9 and 27 Hz indicating a harmonic at 36 Hz and a mild peak between 18 and 27 Hz suggestive of a 45-Hz harmonic (for instructions on how to read a bicoherence plot, see Sheremet et al., 2016). As the power of θ harmonics increases with velocity (Sheremet et al., 2016, 2019a) and a harmonic is defined as being a phase coupled integer to a fundamental rhythm, the increase in the bicoherence between the harmonics with velocity support the interpretation that the low frequencies correlations in Figure 5*D*,*F* are indeed harmonics and not slow γ.

### The redistribution of power in the Fourier spectrum

As described above, power spectra undergo a redistribution of power across frequencies with velocity. θ, the harmonics and the 50- to 120-Hz γ band increase in power, whereas the inter-harmonic intervals decrease in power. Investigations into the relationship between slow γ and velocity have been contradictory (Chen et al., 2011; Kemere et al., 2013; Zheng et al., 2015). As an alternative to the two γ hypothesis, it has been argued that there is a single γ frequency which increases with running speed, resulting in a decrease in amplitude of low-frequencies and an increase at higher frequencies (Ahmed and Mehta, 2012). As described by Ahmed and Mehta (2012), the redistribution of power across frequencies is a consequence of increasing afferent drive onto interneurons (Traub et al., 1996). Specifically, with a low level of afferent input, neurons are weakly entrained exhibiting a range of oscillations. These same neurons become strongly entrained with energy (increasing afferent input) and velocity (Sheremet et al., 2019a). By allowing neurons to interact with each other, a general frequency of entrainment is selected, accompanied by accretion of power in one band with erosion of power in the adjacent bands (Wiener, 1965, 1966; Strogatz, 1994).

**Figure 6.**
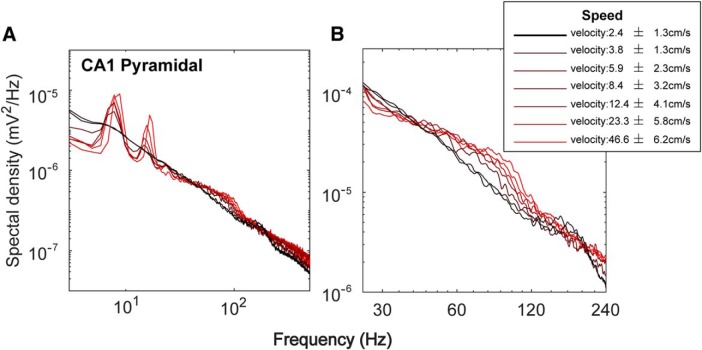
Spectral changes with velocity. ***A***, The PSD across low to high velocities for frequency ranges up to 500 Hz. The power of θ and its associated harmonics increases with faster running velocities. This increase in θ and harmonic power is associated with a decrease in the power in the inter-θ/harmonic frequency bands at higher running speeds. ***B***, The PSD across the 25- to 240-Hz frequency ranges. At higher velocities, power in the 25–50 range decreases. Compare to Ahmed and Mehta (2012; Fig. 2).

We, therefore, examined the relationship between running speed and the power spectra with higher velocity resolution. Figure 6 shows the power spectra between 0 Hz and 1000 Hz and for a narrower frequency range (25–240 Hz). Consistent with previous reports (Czurkó et al., 1999; Terrazas et al., 2005; Sheremet et al., 2016, 2019a), as running velocity increases, there is an associated increase in θ power and its harmonics. As described above, the increase in θ power and its related harmonics was associated with decreases in power in the frequency ranges surrounding θ and its harmonics. Because CA1 neurons are tuned to fire at θ frequency (see Oscillatory modulation of CA1 neuron firing), these observed increases in power at faster running speeds, which also produce higher firing rates of both pyramidal cells and interneurons (McNaughton et al., 1983), can be viewed as the energy into the hippocampus increasing resonance or bringing otherwise incoherent neural activity into coherent, dynamic patterns. A similar idea has been proposed by Traub et al. (1996), in which γ oscillations increase in frequency with increasing drive with the only extension here being that the drive comes in bouts of θ. From this perspective, it becomes odd to describe slow γ as a fundamental oscillation that is maximal in power at low velocities. Accurately, low velocity describes the lowest firing rate condition and a minimal capability of neurons to interact on each other in a manner described by Wiener and Strogatz (Wiener, 1965, 1966; Strogatz, 1994). To consider the loss of power to be data that supports a slow γ rhythm is contradictory as the low velocity/low afferent input state implies that neurons are free to “drift,” and by lacking entrainment with each other, have limited coherence to the LFP. Given this rationale, we explored the frequency of spike modulation, spike-LFP coherence analysis, and a phase-locking (“depth of modulation”) analysis as a function of velocity.

### Oscillatory modulation of CA1 neuron firing

Prior analyses of hippocampal neuron spike times have described modulation by slow γ (Colgin et al., 2009). When examining the preferred phase of the firing of neurons to the hippocampal θ rhythm, however, the spike depth of modulation plots are not pure sinusoids. Rather, there is a definite skew and asymmetry (Skaggs et al., 1996; Quilichini et al., 2010). Furthermore, spectral decomposition of spike trains has revealed the presence of a ∼16-Hz harmonic modulation (Sheremet et al., 2016). In light of the skewness, the concern has been raised that modulation in the 20- to 30-Hz band may not actually be related to slow γ but rather coupled to the asymmetry of θ (Schomburg et al., 2014). Therefore, we compared the phase coupling of neurons to slow γ versus higher-order θ harmonics in the LFP in relation to the animal’s running speed.

**Figure 7.**
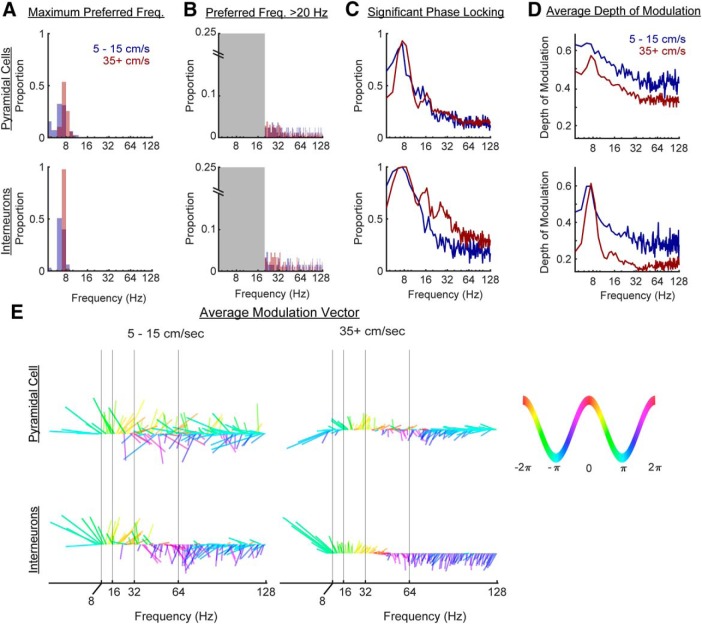
Neuronal depth of modulation can be found for all frequency bands but with little evidence for a slow γ band. ***A***, The maximum preferred phase of neuron modulation was determined for all cells should the neuron have a significant phase locking (as determined by a Rayleigh test *p* < 0.01). When conducting this analysis across a broad band (4–128 Hz) and as a function of velocity, it becomes evident that neurons are primarily modulated by θ. ***B***, However, γ modulation analyses are often conducted without considering the contribution of θ. Therefore, we examined the maximum preferred phase >20 Hz. The major mass of spikes falls near the third harmonic of θ, consistent with the heavy θ modulation seen in panel ***A*** as well as hypothesized by Schomburg et al. (2014). Note the difference in *y*-axes between panels ***A***, ***B***. ***C***, Similar to Schomburg et al. (2014), the proportion of cells that exhibited significant phase locking (as determined by a Rayleigh test *p* < 0.01) was plotted as a function of frequency. However, the primary difference between Schomburg et al. (2014), and the present study was that the aforementioned study implemented wavelet to determine phase, whereas the current study used a narrow band filter (see Materials and Methods). Note that the majority of cells were modulated by the 7- to 9-Hz θ rhythm. Should they exhibit a skewed distribution in their phase preference (Skaggs et al., 1996), then it is conceivable that they would also exhibit significant phase locking to harmonics. ***D***, For each neuron that exhibited significant phase locking to a specific band, the depth of modulation was calculated. In this instance, the sparse firing of pyramidal neurons favors greater depths of modulation at lower velocities (as depth of modulation is the maximum bin value minus the minimum bin value normalized to the maximum; see Materials and Methods). At both low and high velocities, for pyramidal cells and interneurons, the maximum depth of modulation was to the θ. Although there was a large proportion of interneurons modulated by the 21- to 27-Hz harmonic of θ (***C***, bottom), the depth of modulation values in this range approach a minimum, hitting the lowest values in the slow γ range. ***E***, For the significantly modulated neurons, a vector length (depth of modulation) and angle (preferred phase) was determined to allow the calculation of the average modulation vector as a function of frequency. The color and angle depict the phase (see key) while the magnitude of the line is the depth of modulation. At low velocities, there is a high degree of variance across adjacent frequencies which becomes smaller at higher velocities. For of all these plots, while it may be tempting to derive a conclusion for frequencies >16 Hz, we emphasize caution. After the θ range, spike-field coherence values fall well below 0.05 (weak coherence, despite being significant, is closer to an incoherent, random phase assignment than coherence; Fig. 8). Therefore, although it is possible to find significant coupling across the range of bands between 4 and 128 Hz, there is no rationale to suggest that a slow γ modulation exists in the spiking activity of the units.

First, we replicated the analysis of Schomburg et al. (2014), determining the preferred frequency of modulation as well as the proportion of neurons modulated across frequencies (Fig. 7). The only major difference between our approach and that of Schomburg et al. (2014) is that our phase assignment was not conducted via wavelet but through phase assignment relative to a narrow band filter. When considering all frequencies between 4 and 128 Hz, it is evident that most cells exhibit their strongest modulation relative to the 7- to 9-Hz θ rhythm (Fig. 7*A*). Over 90% of interneurons preferred θ at high velocities. Including pyramidal cells, it can be safely stated that nearly every hippocampal neuron is first and foremost modulated by θ. As θ preference eclipses all other frequencies, the explicit consequence is that -during behavior- any other frequency modulation is secondary. Therefore, to explore potential “secondary preferred frequencies,” the same analysis was performed for oscillations >20 Hz (Fig. 7*B*). A small clustering is evident in the 21- to 27-Hz range (although the proportion is much lower than Fig. 7*A*). Parsimoniously this can be explained by harmonic effects: (1) most neurons are strongly modulated by θ (Fig. 7*A*), (2) depth of modulation plots of neurons relative to θ are asymmetric (Skaggs et al., 1996; Quilichini et al., 2010), and (3) harmonic modulation can carry this effect (Schomburg et al., 2014). This approach, however, is “either/or,” not explicitly considering the proportion of the population that is modulated by all frequencies. When asking the question from this angle (Fig. 7*C*), as anticipated, the maximum peak was observed at θ followed by the 14- to 18-Hz harmonic and the 21- to 27-Hz harmonic (interneuron; high velocity). While these values are indicative of significant phase coupling, the average depth of modulation describes how tightly coupled the neuron spikes are to a specific phase (Skaggs et al., 1996; Fig. 7*D*). In the pyramidal cells, low firing rates at low velocity skew the depth of modulation to an artificially high level (that is, bins with a single spike at the trough will cause artificial inflation). With that consideration, the largest depth of modulation resides at θ at both low and high velocity for pyramidal neurons. The interneurons exhibit a similar pattern with the additional peak at the first harmonic of θ at high velocity. These values steadily decrease from θ toward higher frequencies, approaching an asymptotic floor near 32–64 Hz (slow γ).

Finally, as the depth of modulation analysis provides the preferred phase of firing as well as the strength of the modulation, we calculated the average preferred vector of firing for neurons that were significant (as determined by a Rayleigh test *p* < 0.01) as a function of velocity (Fig. 7*E*). As anticipated from Figure 7*D*, the magnitude of modulation was larger at low velocities, although with little consistency in adjacent frequencies. As running speed increases, adjacent frequencies tend to have similar phase preferences. Note well that almost every neuron was modulated by θ, making any other frequency modulation secondary. With respect to other frequencies, as only the neurons that passed a Rayleigh test went into this analysis, it demonstrates that depth of modulation within a singular frequency band is not sufficient evidence in support of a fundamental rhythm. One can always find a significant proportion of neurons modulated to a narrow band. Concerning slow γ, modulation to 25–50 Hz may be a consequence of aliasing and/or coupling to θ harmonics (Schomburg et al., 2014; Sheremet et al., 2016).

**Figure 8.**
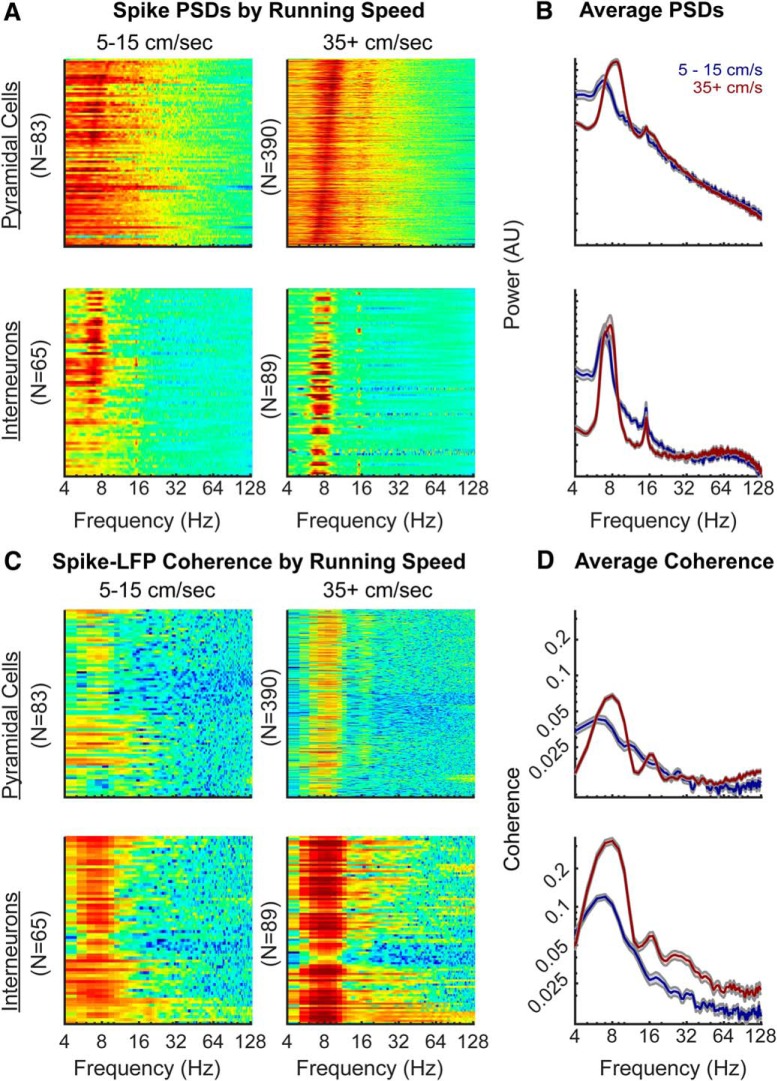
PSD of neuron spike trains and spike-LFP coherence as a function of velocity. ***A***, Individual power spectra for spike trains at low velocity (5–15 cm/s) and high running speed (35+ cm/s; color axes are to the same scale between plot) for interneurons and pyramidal cells. A spike threshold >0.25 Hz was applied to each running bin to ensure that only neurons with a considerable number of spikes were analyzed. The rows are sorted by the maximum frequency between 4 and 12 Hz. ***B***, The average PSDs at low (blue) and high velocities (red); error bars are the SEM (different axes are used to allow the spectral shape to be compared). Note that there is an absence of a spike-frequency peak in the slow γ range although both the interneurons and pyramidal cells exhibit harmonic modulation. Moreover, at high velocity, there is a potential peak in the interneurons that coincides with the traditional γ range of 40–100 Hz (Bragin et al., 1995). Note that the axes are in arbitrary units as power in this circumstance is determined by the number of spikes and thus, each trace was normalized by total power. ***C***, Individual spike-LFP coherence plots for interneurons and pyramidal cells at different running speeds. Once again, the color axis is equal across plots. ***D***, Average spike-LFP coherence by cell type as a function of velocity. While there is a notable difference in overall coherence as a function of velocity, the only peak are at θ, the 14- to 18-Hz harmonic and the 21- to 27-Hz harmonic (prominent at high velocity in the interneurons). Broad modulation in the 25- to 50-Hz band (slow γ) is absent. We emphasize strong caution in interpreting small coherence values (for a similar opinion, also see Buzsáki and Schomburg, 2015). A coherence of 1 would indicate no variance in the phase difference across signals, while 0 would be indicative of a random phase assignment. As the majority of coherence values related to frequencies greater than the 16-Hz θ harmonic fall below 0.05, concluding that units are capable of organizing, or being organized into higher frequencies based on this data are dangerous.

This analysis, however, may not be comprehensive enough to dismiss slow γ modulation. Therefore, we considered the alternative approaches of spike PSD and spike-LFP coherence. First, the point-process spike trains (available on the CRCNS.org website; see Materials and Methods) were sorted into either a low or high-velocity bin, based on the running speed of the rat, and converted into a binary time series. These spike trains were spectrally decomposed to examine the burst frequency modulation (Leung and Buzsáki, 1983; Sheremet et al., 2016). “Power” in this analysis is related to the neurons firing rate which, in the hippocampus follows a logarithmic distribution across the population (Mizuseki and Buzsáki, 2013). Therefore, to ensure that a high firing rate neuron does not skew the overall results, each neuron was normalized by overall power (resulting in power being presented in arbitrary units; Fig. 8). As Bieri et al. (2014) found the most slow γ epochs in the center of a linear track, coinciding with locations of the highest running speed, it may be anticipated that there would be a single peak in the slow γ range of the spike spectrograms at high velocity. Investigations of the spectrograms, however, do not support this idea. Instead, the small observable peaks tend to coincide with θ and the first harmonic. There is no evidence of a slow γ modulation in the neuron spike trains for either interneurons or pyramidal cells. Notably, interneuron firing appeared to be modulated within the 50- to 90-Hz range (Fig. 8*A*,*B*). Finally, we considered that, despite the absence of a depth of modulation and the absence of spikes bursting at slow γ frequency, there might be spike-LFP coherence indicative of an oscillation (Pesaran et al., 2018). Therefore, we calculated spike-LFP coherence by neuron type and as a function of velocity (Fig. 8*C*,*D*).

Coupling was only evident in the θ and harmonic bands. However, as the Spike-LFP coherence falls below 0.05 at frequencies >18 Hz, we emphasize caution as these values lean toward “incoherence” (see Buzsáki and Schomburg, 2015).

Again, there was little in the way that would suggest that slow γ exists. These results beg the question that, if slow γ is absent in the Fourier decomposition, what is responsible for the description becoming dogma? As the initial description of slow γ was predicated on wavelet decomposition, it is tenable that the different methods yield different decompositions with the proposition that one is not maintaining fidelity with the underlying biology.

### Comparison of Fourier, wavelet decomposition, and EEMD

Fourier analysis operates by decomposing a time series into a sum of sine wave oscillations each with a fixed amplitude. There is a fixed decimation in time and frequency resolution in this approach (Fig. 2). A wavelet, on the other hand, has a trade-off between time and frequency such that low frequencies will have a low temporal, high-frequency precision. As the mother wavelet is compressed for higher temporal resolution, it comes at the sacrifice of frequency resolution (Fig. 4). A non-sinusoidal oscillation such as θ, a skewed and asymmetric oscillation at ∼8 Hz, would be represented in the Fourier domain as an 8-Hz oscillation plus the phase locked, integer related harmonics to the fundamental. That is, for the superposition of sine waves to be recombined in a way that reconstructs a non-sinusoidal time series requires the use of harmonics. A wavelet, while also capable of decomposing the same time series, will treat non-sinusoidal oscillations differently. Consider the initial report of slow γ in which the mother Morlet wavelet was set to have a ratio of 7 between the central frequency of wavelet and SD of the applied Gaussian window (Colgin et al., 2009). This setting, based on the Heisenberg time-frequency resolution boxes, is theoretically capable of resolving θ with high-frequency resolution but low temporal precision. For the superposition of wavelets to recreate a nonlinear time series (such as a sawtooth wave), the same method is subject to assigning a high amount of power across a broad range of frequencies in a transient manner. This places the wavelet decomposition method at risk of distorting the frequency-power representation of a non-sinusoidal time series. The 24 Hz and other high-order harmonics of θ would erroneously be decimated into Heisenberg boxes with lower frequency resolution and higher temporal resolution. The consequence being that the 24 Hz, 32 Hz, and higher harmonics of θ could be depicted as a broadband 25- to 50-Hz oscillation. In addition to wavelet analysis, EEMD has been put forward as a method theoretically capable of breaking the LFP into θ and supra-θ bands (Lopes-Dos-Santos et al., 2018). Specifically, Lopes-Dos-Santos and colleagues claim that, following EEMD, the remaining supra-θ signal is “harmonic free.”

**Figure 9.**
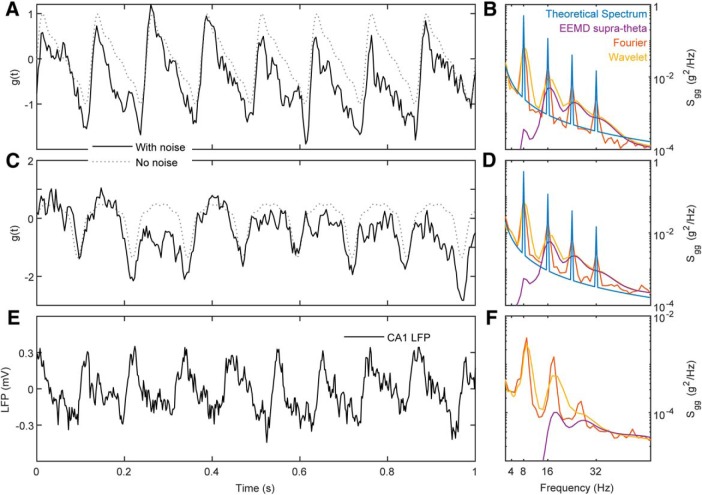
Example of Fourier, wavelet and EEMD analysis on synthetic time series and hippocampal LFP. ***A***, A synthetic time series emulating an LFP recording dominated by an asymmetric θ signal. The signal is constructed from an asymmetric sine function (dashed line; an 8-Hz fundamental oscillation with phase-coupled harmonics), and pink (f^−1.5^) noise (solid line). The inclusion of pink noise provides a more realistic spectrum for comparison to the raw LFP. ***B***, Estimates of PSD using different methods. ***C***, ***D***, A synthetic time series and spectral decomposition as in ***A***, ***B*** but with a different alignment, generating a cnoidal wave. ***E***, ***F***, Actual LFP from the CA1 pyramidal layer and the respective spectral decomposition. As anticipated from the spectral leakage inherent in wavelet decomposition (Fig. 4), neither the wavelet nor EEMD approaches are capable of accounting for higher order harmonics larger than 16 Hz. Rather, the method artificially distributes power over a wide band giving the erroneous impression of a slow γ when there is none in the underlying signal. Once again, we remind the reader that, for wavelet and EEMD, the assignment of variance to any specific frequency is ultimately arbitrary and meaningless.

**Figure 10.**
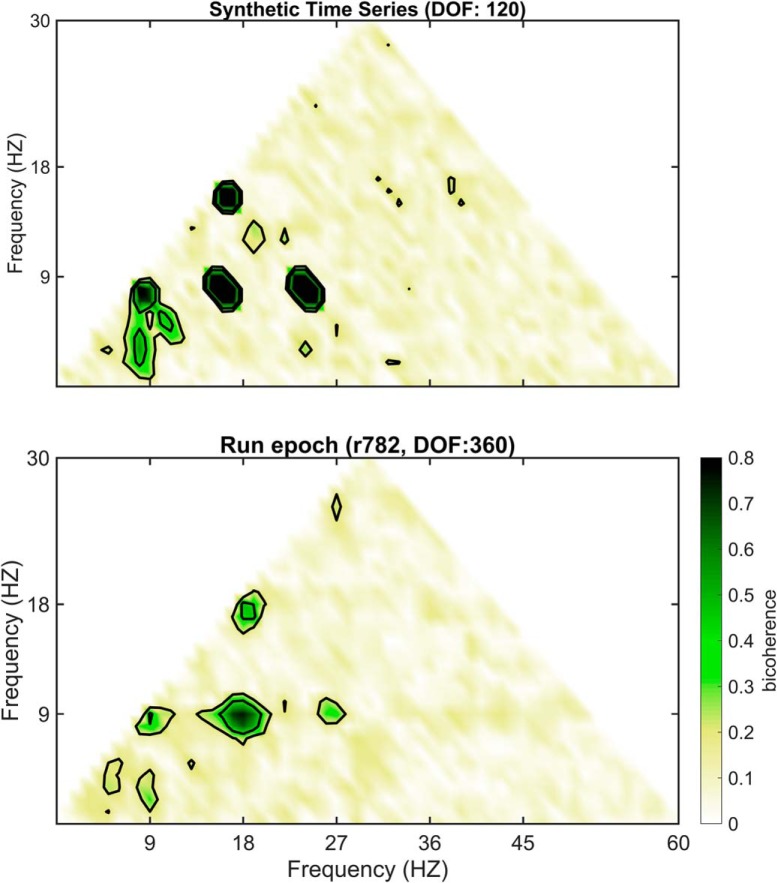
Post-EEMD bicoherence plots demonstrating failure to account for harmonics. In a prior study, it was suggested that “EEMD also provides supra-θ components that are virtually free from harmonic artifacts (Wu and Huang, 2009)…” (Lopes-Dos-Santos et al., 2018, their supplemental p. e3). However, the parent study of Wu and Huang provide no evidence testing the validity of this claim. Therefore, using a synthetic trace as well as LFP data, the post-EEMD processed time series were analyzed with bicoherence. Note that, in both instances, the contour lines outline four distinct regions of triad phase correlations ([8, 8, 16 Hz], [16, 8, 24 Hz], [24, 8, 32 Hz], and [16, 16, 32 Hz]), demonstrative of strong harmonics.

Validity describes the ability of a test or procedure to measure what it claims to measure. As each spectral decomposition method asserts to be capable of decomposing a time series into a frequency-power representation, a simple test is to evaluate their outcomes against a synthetic trace with known frequencies and powers. Therefore, an initial benchmark comparison of Fourier decomposition versus wavelet decomposition and EEMD was conducted using a synthetic time series of pink noise embedded with an oscillation at 8 Hz and harmonics at 16, 24, and 32 Hz (Fig. 9*A–D*). The first synthetic trace was generated by the superposition of harmonics to create a saw-tooth wave while harmonics in the second trace summed to make a completely symmetric, cnoidal wave (demonstrating that simple asymmetry calculations are insufficient to account for harmonics). Notably, the power estimate of the Fourier decomposition closely matched the theoretical distribution. However, as a consequence of the multi-resolution analysis of wavelet decomposition, the power estimate significantly expands the frequency representation of the harmonics to cover a wide band, overlapping with reports of slow γ (Colgin et al., 2009) as well as β (15–30 Hz; Rangel et al., 2015, 2016). Although harmonics were implemented in the construction of these time series, EEMD expressed the same peaks as wavelet demonstrating a failure to remove these components. The three decomposition methods were then applied to the analysis of hippocampal LFP from the CA1 pyramidal cell layer (Fig. 9*E*,*F*). Again, both the wavelet decomposition and EEMD give the impression of convolved harmonics. This is despite the claim that EEMD is free of harmonic effects (Lopes-Dos-Santos et al., 2018). To explore the degree to which EEMD accounts for the harmonics, both the synthetic trace from Figure 9*A* and the LFP data from rat 782 (Fig. 9*E*) were processed with EEMD, and the resulting trace run through bicoherence, capable of detecting harmonics (Aru et al., 2015). The results of Figure 10 reveal multiple interactions at integers of θ. As phase-locked integer oscillations define harmonics, this analysis demonstrates the incapability of EEMD for generating a harmonic free LFP challenging the validity of the initial claim. Furthermore, the implementation of wavelet with a ratio of 7 between the central frequency of wavelet and SD of the applied Gaussian window (Colgin et al., 2009) “convolves” over the higher-order harmonics (plausibly accounting for why studies using wavelet decomposition rarely identify a harmonic above 16 Hz). Importantly, this convolution loses fidelity to the underlying oscillation generating an invalid power-frequency plot, that is, does not match the true relationship between frequency and power in the synthetic trace.

**Figure 11.**
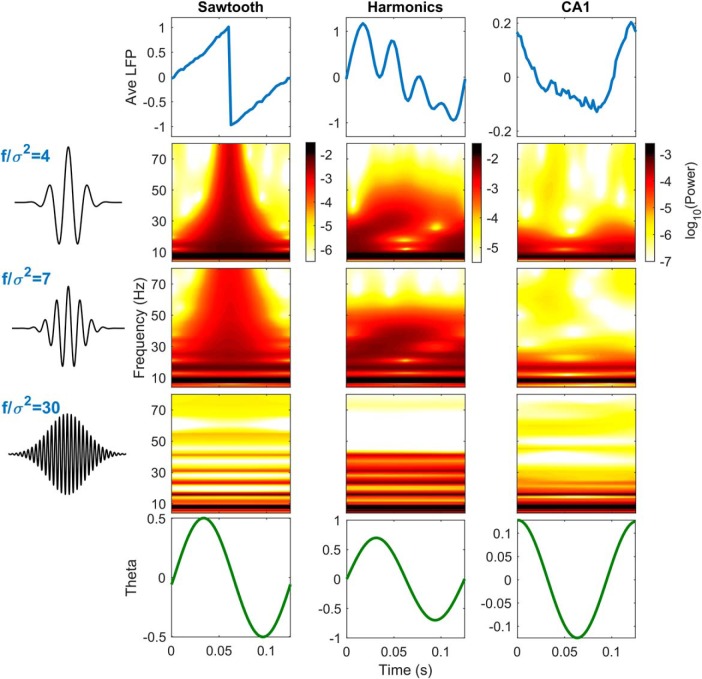
Scalograms for synthetic time series and LFP recordings in the CA1 pyramidal cell layer using different wavelet parameters. The averaged time series for one θ period (8 Hz) and wavelet scalograms were obtained for three time series: (1) synthetic sawtooth waves (left to right, the 1st column); (2) time series with fundamental 8-Hz oscillation and its harmonics (16, 24, 32, and 40 Hz; the 2nd column); (3) LFP recorded in CA1 pyramidal cell layer (the 3rd column). Synthetic time series have a total length of 10 s and were added with a pink background noise. LFP recordings were selected during high speed running with a total length of 30 s. The averaged time series were obtained by averaging over θ periods (top row). The wavelet scalograms for averaged time series were computed with three different wavelet transforms (the three middle rows). The Morlet wavelet family is characterized by the constant ratio between the central frequency of wavelet 2 SD of the applied Gaussian window. Therefore, the results for three different Morlet wavelets are presented. Averaged time series were filtered within θ band (7.5–8.5 Hz) which represent the θ phase (bottom row). A sawtooth wave is decimated into an infinite series of harmonics in Fourier decomposition (https://en.wikipedia.org/wiki/Sawtooth_wave). The lowest ratio of 4 optimizes for temporal resolution over frequency resolution, whereas the highest ratio of 30 has an increased frequency resolution at the expense of temporal resolution with results that are more in line with Fourier analysis. This is depicted in the sawtooth scalograms as a temporally localized, wide frequency band event in the Morlet ratio of 4 but as frequency specific, time diffuse bands in the Morlet ratio of 30 condition. Note that the latter condition resolves harmonics. This effect is also evident when combining an 8-Hz oscillation with its first few harmonics (16, 24, 32, and 40 Hz) through additive synthesis in developing a sawtooth wave. The Morlet ratio of 4 convolves over the higher-order harmonics, giving the impression of a 25- to 50-Hz cross-frequency interaction, whereas the wider ratio of 30 resolves each individual component. These effects carry over to LFP data collected from the CA1 pyramidal layer. The middle ratio of 7 was selected to match the methods of Colgin et al. (2009). Note that the parameters for the ratio of 7 are more closely aligned with the ratio of 4 in that the harmonics are heavily convolved across a wide frequency band, giving the impression of a wideband oscillation when none exists.

With this concern, we proceeded to revisit the wavelet scalogram to investigate the interaction between γ frequency and the θ oscillation (Colgin et al., 2009) as well as determine whether a more reasonable decomposition could be achieved with wavelet. This was first conducted for synthetically generated data (a sawtooth wave and an oscillation with integer locked harmonics) as well as data collected from CA1 pyramidal cell layer (Fig. 11). Often understated is that the selection of Morlet wavelet parameters will dramatically alter the decomposition. The mother Morlet wavelet is characterized as the frequency of a carrier oscillation and the deviation of the Gaussian envelope. This parameter effectively alters the trade-off between the time-frequency resolution. Therefore, we also investigated the ability of each Morlet ratio to resolve the underlying frequencies in relation to time. For the scalogram decomposition of the sawtooth wave, the low Morlet wavelet ratios (4 and 7) capture the instantaneous change in amplitude by distributing power across a wide frequency band. The higher Morlet wavelet ratio (30) effectively does the reverse, capturing the nonlinear oscillation as a series of harmonic oscillations that are well resolved in frequency. While this is a simulation to an extreme derivative, the time-frequency representation of the synthetic harmonic series (8, 16, 24, 32, and 40 Hz), generating a mild sawtooth wave is nearly identical to the initial report of slow γ in the CA1 pyramidal cell layer for the low ratio wavelets (Colgin et al., 2009). Note that in this simulated trace, the highest frequency is 40 Hz, whereas the low ratio wavelet scalograms skew the power toward 60 Hz. The higher ratio wavelet, however, resolved each of these individual harmonics at the cost of assigning the power to a specific time. Nevertheless, as the synthetic harmonics are convolved by the lower ratio wavelets (7 being the one implemented in the initial description of slow γ) to give the impression of a broad slow γ band, LFP collected from the CA1 pyramidal layer is subject to an identical distortion (Fig. 11). When comparing Figure 11 to the initial report of slow γ (Colgin et al., 2009, their Fig. 1*E*) there are dramatic differences in power across frequencies. Although the power spectrum has a relationship such that low frequencies have high power and high frequencies have low power (often described as the A = f^-α^ slope), the scalograms in Colgin et al. (2009) suggests that the power in the >100-Hz band is significantly larger than the 50-Hz band. This is due to the application of a pre-whitening filter resulting in the caveat that “…power magnitudes depicted in these illustrations should be ignored since they do not accurately reflect the actual size of waveforms in the original local field potential recordings” (Colgin et al., 2009, their supplementary information, p. 38). While whitening was implemented to visualize power at higher frequencies, the initial depiction justifying the division of slow γ was acknowledged to be an inappropriate representation of the raw data. This issue is compounded when considering the validity of the wavelet parameters selected for decomposition.

**Figure 12.**
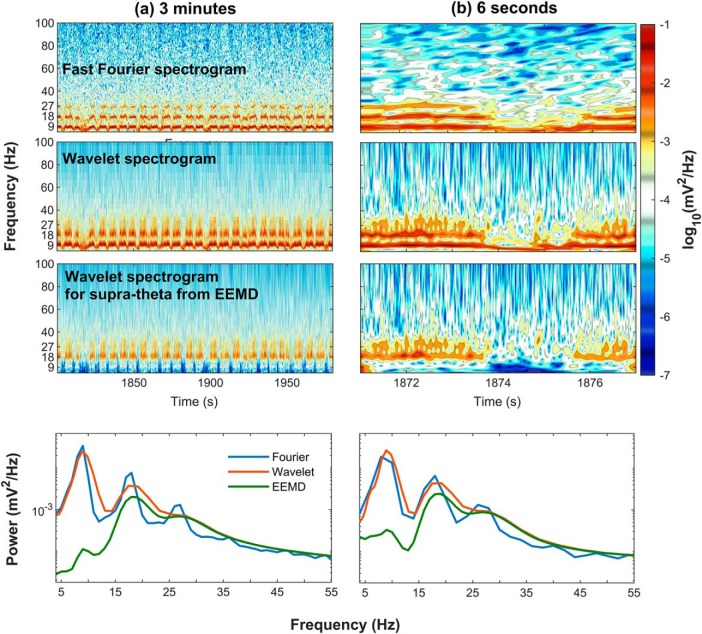
Spectrograms and power spectra obtained from recordings while rats were running on a maze. Spectrogram and power spectrum were computed for a long run epoch (column ***a***, 5 min) and a short epoch (column ***b***, 6 s). For each epoch, the spectrograms were estimated with three methods: (1) Fourier transform (1st row) with a window length of 1 s and window increment of 0.1 s; (2) wavelet transform (2nd row) with Morlet wavelet; 3) wavelet spectrogram of supra-θ signal (3rd row). The supra-θ signal was obtained from EEMD with noise level equaling to 0.5 total variance and ensemble number equaling to 200. The supra-θ was defined as the sum of decomposed modes whose central frequencies were larger than 12 Hz. The wavelet spectrogram of supra-θ was computed with the method described above. Power spectra were computed by averaging the spectrogram over time (bottom row). For both epochs, the Fourier transform identifies θ and high order harmonics while wavelet analysis tends to resolve a θ rhythm and a wide-band frequency component (16–30 Hz). By considering this band as independent from θ in this manner gives the unintentional representation that there are “bursts” of γ. Stated differently, the 24-Hz oscillation is inherently dependent on the nonlinearity of θ (“sawtooth” shape of θ) and thus is incorrectly decomposed by the wavelet.

As the results of wavelet and EEMD are often portrayed in terms of a time-power-frequency representation, we furthered the comparison using two different time scales of analysis (Fig. 12). Both in the long (3-min Fourier transform) and short (6 s Fourier transform), there is the definite presence of a third-order θ harmonic (∼24–27 Hz) and a trace of a fourth harmonic (∼32–36 Hz; Fig. 12, top panels). However, as anticipated from the Heisenberg boxes (Fig. 4), the wavelet decomposition as implemented in previous studies results in a convolution and apparent intermittency of the ∼24- to 27-Hz frequency band that is particularly evident at the 6-s time scale. Because the 24- to 27-Hz component is a direct consequence of θ being skewed and asymmetric (for example, more “saw tooth” shape than a sinusoid; Buzsáki et al., 1983, 1985; Terrazas et al., 2005; Sheremet et al., 2016), this frequency is inherently coupled to the fundamental 8-Hz rhythm (that is, as stationary as θ; not a transient event). The wavelet decomposition as implemented in prior studies, however, artificially detaches the third harmonic from the fundamental by using a different decimation of time and frequency (Fig. 2). This convolution and intermittency offers a strong resemblance to experiments that report different θ cycles exhibiting unique γ signatures (Colgin et al., 2009; Bieri et al., 2014). To an extreme, using EEMD, it has been argued that θ can mix and match different γ frequencies (Bagur and Benchenane, 2018) in support of different memory processes (Lopes-Dos-Santos et al., 2018). Therefore, we also replicated the methods of Lopes-Dos-Santos et al. (2018). Notably, comparing both the long and short temporal epochs of wavelet decomposition of the “supra-θ” reveals the distorted remnants of the second harmonic of θ as well as the third harmonic (Fig. 11). Finally, the summary power spectra for each method is presented (Fig. 11, bottom panels) demonstrating the inability of either wavelet, or EEMD followed by wavelet to resolve the θ harmonics. This perspective gives rise to another issue, that is, although the harmonics of θ effectively mirror the stationarity of θ, the varying time-frequency boxes of wavelet give the invalid impression of transience.

**Figure 13.**
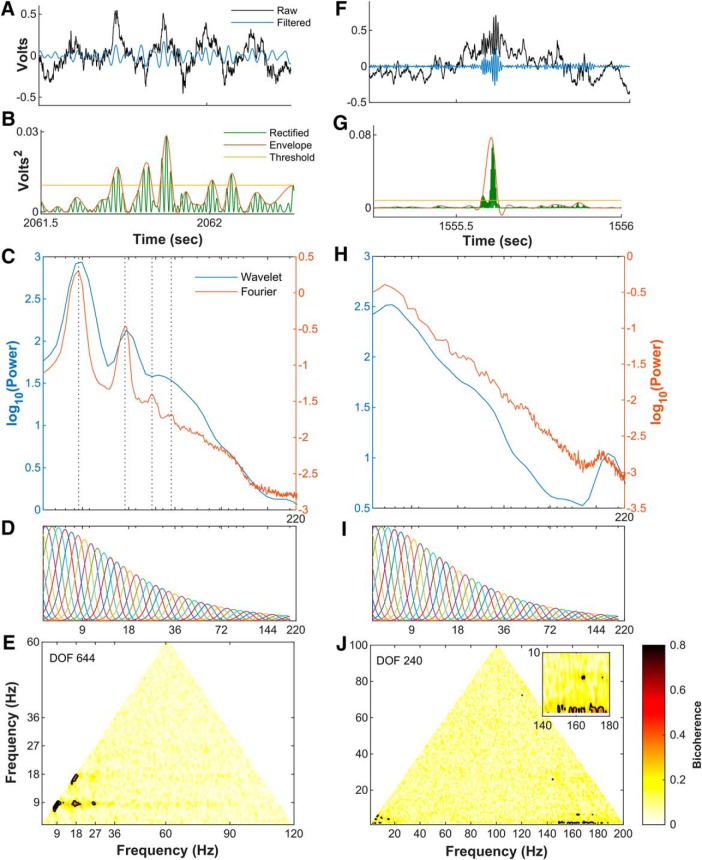
Power based detection of ripples and slow γ and the corresponding Fourier and Wavelet decompositions. ***A***, Raw and filtered (25–50 Hz) trace of pyramidal layer LFP. ***B***, High-amplitude 25- to 55-Hz events were detected by calculating the mean and SDs of the envelope of the squared filtered trace. ***C***, For each instance in which the slow γ power exceeded 2 SDs above the mean power, a 2-s window was extracted and processed using either Thomson’s multi-taper method or wavelet decomposition using the same approach as Colgin et al. (2009). Interestingly, the wavelet decomposition reveals a slow γ bump (suggesting that wavelet would concur that these are slow γ epochs), whereas the Fourier decomposition shows multiple bumps indicative of harmonics. ***D***, Frequency range of the individual wavelets implemented in Colgin et al. (2009). As the frequency increases so does the range of an individual wavelet which is responsible for the redistribution of power across a broad band. ***E***, Bicoherence decomposition of the epochs in which the 25- to 55-Hz power exceeded 2 SDs above the mean. The significant regions at 18 and 18 Hz and 9 and 27 Hz indicate an interaction with the 4th, 36-Hz harmonic (Sheremet et al., 2016). Therefore, this demonstrates based on the parameters used in prior publications, the wavelet approach is convolving over harmonics of θ to erroneously give the impression of a slow γ bump. ***F***, Raw and filtered (120–250 Hz) trace of pyramidal layer LFP. ***G***, Ripple events were detected when the envelope of the rectified trace exceeded 2 SDs above the mean. ***H***, Analyzing each detected instance of a ripple in a 2-s window reveals a significant power in the ripple frequency demonstrating that it is possible to decompose transient events such as high-frequency ripples. ***I***, Same as ***D***. ***J***, Bicoherence decomposition of the detected ripple epochs reveals an interaction between low-frequency events at ∼2–10 Hz and a 150- to 180-Hz band (see inset). This interaction is most likely a consequence of coupling between ripples and sharp wave related deviations.

### Slow γ and the issue of transience

The slow γ (25–50 Hz) and “fast γ” (60–100/140 Hz) oscillations have often been discussed as being non-stationary and transient (Bieri et al., 2014; Zheng et al., 2015; Lopes-Dos-Santos et al., 2018). Therefore, it is plausible that slow γ is so transient as to be averaged out by Fourier decomposition (although leaving one to wonder why frequencies between 50–120 Hz and ripples are reliably resolved by Fourier decomposition; see Buzsáki et al., 2003 and Johnson and Redish, 2007). While wavelet is capable of detecting the presence of a transient high-frequency event, it is liable to distort the frequency of the same event (as demonstrated in the analyses on the synthetic traces above); power and frequency range quantification will have diminished meaning. To this point, we have demonstrated that wavelet as traditionally implemented is subject to this deformation, but we have not considered the possibility that transient 25- to 50-Hz high power bursts exist in the LFP.

Therefore, to cross-validate the detection of slow γ events with quantification, we implemented a simple filter method to identify transient events. First, the 25- to 55-Hz filtered trace was squared (rectified; Fig. 13). Next, the mean and SD of the envelope of the rectified trace was calculated. As instances in which power (amplitude squared) exceed 2 SDs above the mean has been reported to optimize between detecting slow γ events and rejecting noise (Zheng et al., 2015), we mirrored this approach. Thus, although the statistical characteristics of the LFP on the whole change across long time scales, by triggering to a known event, the LFP can be treated as locally stationary, which in turn allows the spectra to be realized (Pesaran et al., 2018). Importantly, this method has also proved sensitive enough to detect the transient low amplitude, high-frequency ripples (Maurer et al., 2006; Skaggs et al., 2007; n.b., the amplitude of ripples are lower than lower frequency events).

Analyzing 2-s epochs centered on high power 25- to 55-Hz events using the multi-taper and previous implemented wavelet methods resulted in two contradictory representations. The Fourier based multi-taper decomposition peaks at a 24 Hz and potentially a 32-Hz harmonic of θ which are absent in the wavelet decomposition. In their place is a single, broad band 25- to 55-Hz bump indicative that these events would most likely overlap with the same epochs identified using the method of Bieri et al. (2014). Note that neither of these two decompositions offer a “mixed representation” where both harmonics and slow γ are present. Rather, the outcome is dichotomous and dependent on the decomposition. to determine which representation is true to the biology, the high-power epochs were then processed through bicoherence analysis to test for harmonics (Aru et al., 2015). It is worth emphasizing that the wavelet power spectra give the impression that these are indeed high power, 25- to 50-Hz events. In the bicoherence plot, however, there is a peak interaction between 17 and 17 Hz as well as between 8.5 and 25.5 Hz indicative of θ harmonics as high as 34 Hz. Therefore, while the method of detecting slow γ is “corroborated” by the wavelet decomposition, the high-power 25- to 55-Hz events are in fact a biological consequence of higher order θ harmonics.

To demonstrate that this method is indeed capable of resolving transient events, the same approach was applied to a 120- to 250-Hz filtered trace. Despite the intermittent nature of ripples, both wavelet and multi-taper revealed a peak in the ripple band although the wider Heisenberg uncertainty boxes of wavelet distributes the power of ripples over a wider range. Furthermore, the bicoherence analysis reveals interactions of the ripple frequency with a low frequency event. Therefore, in our data, it becomes evident that the spectral decomposition methods selected determine whether slow γ or θ harmonics are observed.

## Discussion

Accuracy describes the proximity of a measurement to the quantity’s true value, whereas precision is the degree to which the method yields a consistent result under unchanged circumstances (ISO 5725-1, 1994; VIM, 2004). Evidence in support of slow γ has come in the form of the ability of wavelet to reliably achieve the same answer. The present manuscript, however, provides evidence that EEMD and wavelet-based methods, using common parameters, inaccurately represents the LFP. Using data generously provided by the Buzsáki laboratory, we observed cross-frequency coupling between θ, θ harmonics, and a unitary 50- to 120-Hz γ band (also see Sheremet et al., 2019a). In the power spectra from the CA1 pyramidal layer LFP, there was no detectable peak in the slow γ range. These results are largely in agreement with prior publications implementing Fourier decomposition describing θ, harmonics of θ and a broad, high-frequency unitary γ band in CA1 (Buzsáki et al., 2003) and CA3 regions (Johnson and Redish, 2007). The absence of slow γ in CA3 is notable as this region is hypothesized to be the communication conduit of slow γ to CA1 (Colgin et al., 2009). Fourier does not yield the same values as wavelet.

However, the absence of a peak is not indicative that a fundamental rhythm is absent. A coherence may exist between the LFP and spiking activity of single units (Pesaran et al., 2018). Using data available on the CRCNS.org repository provided by the Buzsáki laboratory, we analyzed the spike frequency of neurons, the spike-LFP coherence, and the depth-of-modulation characteristics. When the firing frequency of spikes were analyzed, there was modulation of both principal cell, and interneuron firing rate by θ and its first-harmonic at low and high running speeds, but no modulation at the 25- to 50-Hz band was observed. These results were buttressed by the spike-LFP coherence analysis, again failing to resolve a slow γ interaction. Finally, we analyzed the spike modulation by different oscillatory frequencies within the hippocampus. Notably, there was modulation across all frequencies, although θ modulation eclipsed all others. As modulation is the default, finding significant modulation with a specific frequency band does not qualify as support for a fundamental rhythm.

In our past studies (Sheremet et al., 2016, 2019a) as well as the present manuscript, we have thrice failed to replicate the initial observation of a 25- to 50-Hz oscillation in the CA1 pyramidal layer with either bicoherence analysis or Fourier decomposition (Colgin et al., 2009). If neither spike modulation nor Fourier decomposition provides support for the slow γ hypothesis, then what is responsible for the fervent dogma for the phenomenon? Here, we demonstrate that wavelet ratio of 7 reliably decimates a time series into an inaccurate representation.

Fourier decomposition operates by decomposing a time series into sine waves, allowing the construction of a power spectrum by which each frequency is assigned a unitary amplitude value. There is a fixed ratio between time and frequency where increasing frequency resolution comes at the cost of decreasing temporal resolution and vice versa. Wavelet, on the other hand, uses a varying time-frequency decimation in which there is a progressive increase in temporal resolution as frequency increases. The distortion related to the non-uniform time-frequency decimation was noted early in the application of wavelet to neuroscience data. Tallon-Baudry et al. (1997) investigated the 30- to 70-Hz cortical γ rhythm, using the same wavelet ratio of Colgin et al. (2009) of 7, describing the trade-off: “At 20 Hz, this leads to a wavelet duration … of 111.4 msec and to a spectral bandwidth … of 5.8 Hz, and at 100 Hz to a duration of 22.2 msec and a bandwidth of 28.6 Hz” (Tallon-Baudry et al., 1997, p. 724). The spectral support for this wavelet generates an overlap between the 21- to 27- and the 28- to 36-Hz range such that the power for one θ harmonic will bleed into the other (Fig. 4). Assuming 125 ms for a single θ cycle, the precision of temporal locking measured in this manner will bleed power across ∼78–106 ms for 25–50 Hz, or >180°. Despite these shortcomings, wavelet was favored for its ability to identify the temporal onset of a transient event (Tiitinen et al., 1993; Sinkkonen et al., 1995). The hippocampal γ oscillation could theoretically be considered a transient event necessitating the use of wavelets. A problem arises, however, if the same method used to detect the events is also used to quantify the frequency should it have a trade-off in one dimension (high temporal precision) versus another (low precision in frequency). The non-uniform time-frequency representation distributes the power over a wide frequency band depending on the wavelet parameters used. As demonstrated in Figure 11, the methods using the parameters in the initial description of slow γ are incapable of resolving harmonics in a synthetic trace. Rather, power is distributed across a wide band, giving the impression of a non-existent 25- to 50-Hz oscillation. Note that it is possible to construct an appropriate wavelet that can resolve the harmonics (Fig. 11). More recently, the application of EEMD to the LFP in the CA1 pyramidal layer suggests that there are two γ bands <50 Hz in frequency (Lopes-Dos-Santos et al., 2018). Although it was argued that EEMD could remove harmonics, no verification was presented. When tested against synthetic traces and the LFP, EEMD failed to remove the harmonics (Fig. 10). As the EEMD results were then passed to an aggressive wavelet convolution (Fig. 3) in Lopes-Dos-Santos et al. (2018), the power in the θ harmonic range was distributed across 25–50 Hz, giving the erroneous impression of low-frequency γ bands. These issues extended into the time-frequency spectrograms in which a fourth harmonic was detectable by Fourier, but not resolved with wavelet or EEMD (Fig. 12). Finally, we considered the possibility in which slow γ is transient to the point of being averaged out by Fourier decomposition. Epochs were analyzed in which the power of either ripples or slow γ exceeded 2 SDs above the average power, allowing potentially non-stationary events to be treated as locally stationary (Pesaran et al., 2018). While Fourier based methods revealed a peak in the ripple frequency in the decomposition, only harmonics were present in the epochs in which the 25- to 55-Hz band exceeded 2 SDs above the mean power. The presence of harmonics was verified with bicoherence analysis. Wavelet run on the same epochs, however, convolved over the harmonics and gave the impression of a slow γ band.

Moving forward we can offer two suggestions. First, as velocity is known to increase θ harmonic power (Sheremet et al., 2016, 2019a), future approaches into spectral decomposition should be parameterized by rat-running speed or, at the least, sorted by overall variance (Sheremet et al., 2019b). Second, as analytical toolboxes become more ubiquitous, caution has been emphasized that the user needs to be aware of the caveats or otherwise risk generating mistakes (Marder, 2015). In light of this, we underscore the importance o*f* testing the decomposition method against a synthetic trace with harmonics as well as Fourier decomposition (confirming the validity).

This manuscript questions the validity of analytical approaches used to describe a broadband 25- to 50-Hz slow γ oscillation in the CA1 pyramidal layer as a fundamental hippocampal oscillation. Here, we demonstrate that different spectral decomposition techniques yield different representations of the data. First, while we can replicate the initial findings of Colgin et al. (2009) and Lopes-Dos-Santos et al. (2018), these decompositions are at odds with the Fourier decomposition. Second, wavelet analysis is unstable to the change of parameters and in fact, drifts toward the Fourier representation when the wavelet is optimized for frequency resolution. Third, Fourier decomposition does not offer a realization that is supportive of the slow γ observation, but rather harmonics of θ. Finally, it needs to be re-emphasized that none of these methods offered a mixed representation of slow γ superimposed onto the higher order harmonics of θ. In the instance that they coexist, Fourier spectrum would be able to represent both rhythms. However, this is not the case for the present manuscript. The dissociable outcomes of slow γ and θ harmonics were directly related to the decomposition method and parameters selected (as evidenced in the analysis of the synthetic trace). As each decomposition gives a different representation of the underlying time series, they all cannot be high-fidelity representations of the actual biology. Without having access to the data of Colgin et al. (2009) and Lopes-Dos-Santos et al. (2018), we are incapable of determining whether their reports are consistent with Fourier decomposition. Although the absence of slow γ in the spectral decomposition is complimented by the lack of neuron action potential modulation in the 25- to 50-Hz band, it is necessary to reiterate that the current study does not refute that the brain is capable of exhibiting slow γ. The detection of such oscillatory events in the studies where wavelet or EEMD analyses were implemented with improperly tuned parameters, however, cannot be used as evidence of the existence of a 25- to 50-Hz oscillation that is independent from other frequency bands and meaningful for behavior.

To our knowledge, this is the first attempt to disambiguate slow γ from θ harmonics. If a slow-γ rhythm exists, this analysis points, in the least, to the need of disambiguating it from higher order θ harmonics. After accounting for the harmonics of θ here, there was little reason to support the slow γ hypothesis. Previous work has suggested that the decrease in power in the 25- to 50-Hz range that occurs as a function of velocity is in support of the multiple γ hypothesis (Fig. 6). While this figure could be interpreted as support for two dissociable γ rhythms, change in 25- to 50-Hz power with velocity is, in fact, equivocal. There have been reports of decreasing slow γ power with velocity (Kemere et al., 2013), no change in power with velocity (Zheng et al., 2015), or increase in power with velocity (Chen et al., 2011). Bieri and colleagues did not run an explicit analysis, but their highest proportion of slow γ events occurred in the middle of the track where the running speed was highest and fewest detected events occurred at the ends of the track where velocity was the lowest (Bieri et al., 2014, their supplemental data), suggesting a positive relationship between power and velocity. As an alternative hypothesis, Ahmed and Mehta (2012) argued that there is a single γ frequency which increases with running speed, resulting in a decrease in amplitude of low-frequencies and an increase at higher frequencies. They interpreted their findings as being consistent with the work of Traub et al. (1996), who observed an increase in γ frequency as a function of feedforward drive. To our knowledge, this is the first description of the hippocampal power spectra undergoing a redistribution of power across frequencies as a function of afferent input.

In accord with this, we observe a spectral reorganization in which some bands increase in power while others decrease as running speed increases (Sheremet et al., 2019a, b). The drops in power occur between 1 and 6, 10 and 14, 19 and 23, and 25 and 40 Hz (Figs. 5, 6). This type of spectral reorganization has been described before and has a long history. Norbert Wiener observed an increase in the 10-Hz α power that was associated with dips (loss of power) in the adjacent frequencies (Wiener, 1965, 1966). The insight was that the global oscillation in the EEG arose from the collective action of the population. As described by Steven Strogatz in his computational model of the phenomenon is: “…that the oscillators interact by pulling on each other's frequencies - if an oscillator is ahead of the group, the group tends to slow it down. If it is going too slowly, the group tends to speed it up. In this way the population of oscillators can achieve a collective enhancement of precision.” (Strogatz, 1994, p. 122). Oscillations of the hippocampus do not reside within a single cell or a pair of cells but across the entire population. As the hippocampus becomes more excitable with running speed, the oscillatory dynamics achieve a “collective enhancement of precision” (for a similar perspective, see Churchland et al., 2010). Therefore, the power in 25- to 50-Hz frequency band at low running speed is a consequence of incohesive oscillatory dynamics among the neurons when not entrained (making the claim that slow γ is a fundamental rhythm dubious; oscillations tend to be equated to entrainment, not incoherence). Once input is large enough, the oscillators fall into an en masse alignment, enhancing the power in some frequency bands at the expense of others.

Of course, the question arises: What empirical data would be needed to support an assertion of slow γ?. The consideration of this question raises the core issue: what is a rhythm? Traditionally, a “rhythm” has been identified visually, as peaks above some background level. For instance, in the construction of the PSD, θ exhibits a clear peak above the 1/f^α^ slope. Identifying peaks at higher frequencies are more difficult and a clear deviation from 1/f^α^ for a broad 50- to 120-Hz γ often only becomes evident at high running speeds (Fig. 6) when neuron firing rate is elevated, and there is more energy in the hippocampal network (Sheremet et al., 2016, 2019a). The implied assumption is that the peaks above noise (that is, the rhythms) are the “meaningful” signal and the background is “meaningless,” that is, noise. While the signal/noise dichotomy is ubiquitous in physics, it serves the purpose of defining the scale of interest, by acknowledging that the scale and physics governing the signal are fundamentally different from that of the noise. Fourier and wavelet decompositions both yield representations in which there is power across all frequencies (Fig. 9). Thus, if decomposition reveals power across all frequencies, is it meaningful to segregate any frequency band and ascribe it meaning without understanding how it is different from any of the other frequencies in terms of the network dynamics and biophysical mechanisms that underlie that frequency? From an alternative perspective, consider that a single dynamic process in the network is capable of casting multiple frequencies (akin to an EKG). In this sense, using a correlative approach to divvy up frequencies relative to behavior is not likely to provide much in the way of insight with respect to how the brain organizes behavior (Buzsáki, 2005). In fact, the notion of a multiplexed “spectral fingerprint” (Canolty et al., 2010; Siegel et al., 2012; Knight and Eichenbaum, 2013; Watrous et al., 2013; Akam and Kullmann, 2014; McLelland and VanRullen, 2016) in which different frequency bands independently support unique aspects of cognition should be rigorously tested alongside the alternative model of a spectral energy cascade (Sheremet et al., 2019b).

It should be noted here that, from the biological perspective, the concept of multiplexing is physiologically untenable as it is inaccurate to assume that the LFP recorded near the soma of CA1 pyramidal cells is a linear mirror of the LFP from its afferent inputs. The relationship between the LFP and the source is not this simple or straightforward (Herreras, 2016). With respect to the “routing hypothesis” (Colgin et al., 2009), it needs to be understood that CA3 inputs terminate into the stratum radiatum (more proximal to the soma) whereas entorhinal synapses terminate on the distal dendrites in the lacunosum-moleculare (Amaral and Witter, 1989; Witter and Amaral, 2004), further from the CA1 stratum pyramidale. However, dendrites operate as low pass filters (Golding et al., 2005) in which synaptic input may be γ paced at the dendrites, but become a bolus of low-frequency (θ) input at the soma (Vaidya and Johnston, 2013). Rather than routing information, the Schaffer collateral and perforant pathway inputs can be parsimoniously described as providing energy into CA1. These synaptic inputs are going to interact, as any depolarization or hyperpolarization of the cell is going to affect the ionic driving force. In other words, Schaffer collateral input will influence entorhinal cortical input and vice versa. Thus, γ in the CA1 pyramidal layer is not a mirror of its afferent input, but a consequence of local interactions between pyramidal neurons and interneurons (Marshall et al., 2002; Maurer et al., 2006). Should excitatory drive into CA1 come in bouts of θ, then increasing the power of the θ-paced afferent input promotes more local γ rhythmicity (describing an energy cascade).

The spectral energy cascade hypothesis (Bak et al., 1987; Buzsáki, 2006; Sheremet et al., 2019b) proposes that there is a nonlinear interaction across scales, resulting in both energy exchange and phase coupling between different scales (frequencies). With respect to hippocampal LFP, the amplitude and phase of γ will be inherently coupled to the amplitude and phase of θ. The amplitude and phase of all meaningful high-frequency rhythms will be coupled to higher power/lower frequency rhythms. Should this be true, there is corollary evidence that the θ rhythm provides the necessary energetic drive for neurons to engage in γ rhythmicity. A big peak in θ means “big energy” to lower amplitude, higher frequency rhythms, including γ. Continuing this line of logic, as noted above, peak and phase are inherently coupled such that the highest amplitude is defined as phase zero. From the energy cascade perspective, where θ forces γ, why is it surprising that the phase of θ exhibits a relationship to the power of γ? One would suppose that external, θ-paced input from the entorhinal cortex and septum would trigger a cascade of higher frequency events in the hippocampus and stronger input results in a larger amplitude γ. Thus, the rhythms of the hippocampus should not be considered to have orthogonal relationships with each other. Instead, the most straightforward description is that all rhythms in the LFP are interdependent, coupled by the cascade of energy across spatial and temporal (frequency) scales.

Taken together, the data presented here provide a parsimonious explanation to why wavelet and EEMD analysis results in the appearance of a slow γ band. For both decomposition methods, this involved the parameters that are used to optimize temporal resolution at the cost of frequency resolution. Admittedly, this study cannot claim to have done an exhaustive investigation of every brain region, the entire parameter space, and all possible decomposition methods. Thus, it is our intention that this work will serve as the impetus for future research that includes additional decomposition tools and brain regions.

## Synthesis

Reviewing Editor: Mark Baxter, Mount Sinai School of Medicine Decisions are customarily a result of the Reviewing Editor and the peer reviewers coming together and discussing their recommendations until a consensus is reached. When revisions are invited, a fact-based synthesis statement explaining their decision and outlining what is needed to prepare a revision will be listed below. The following reviewer(s) agreed to reveal their identity: L. Stan Leung.

This report addresses the possible problem with interpretation of a hippocampal LFP frequency band, the slow gamma of ~20-55 Hz, as an independent rhythm rather than high-order theta harmonics. For artificially generated time signals consisting of only theta and its high-order harmonics, the authors convincingly showed that while short timescale Fourier analysis clearly shows theta harmonics, wavelet and EEMD decomposition display broad power (in spectrograms or scalograms) in the 20-55 Hz range (not clearly identified as harmonics). The authors also provided a clear mathematical reason for such a dispersion, since wavelets of higher order have short time duration and wide frequency dispersion (essentially the Heisenberg uncertainty principle). There can be no dispute that theta harmonics will invade into the slow gamma range, with dispersed peaks. The authors also provided evidence from hippocampal LFPs sorted according to velocity of movement of behaving rats. As theta frequency and power increased with velocity, the power of 20-55 Hz power in the hippocampal LFP actually decreased, unitary 22-55 Hz power of pyramidal cell unitary firing showed no change, while power of interneuronal firing increased in the ~60-100 Hz range. This shows that hippocampal slow gamma varied in a direction opposite to theta harmonics, and that slow gamma responded differently from the theta harmonics.

Major concerns:

1) There is ample evidence that there is a slow gamma frequency band, distinct from the theta harmonics. One piece of evidence is that urethane anesthesia decreased the theta frequency and harmonics, and yet increased slow gamma power (Buzsaki et al., 2003). The authors provided another in terms of slow gamma power decline while theta harmonics increase. For experimental data, while knowing that clear theta harmonics (revealed by power spectra) invade into the slow gamma frequency range, this report does not give an evaluation of how serious this problem could be using wavelet analysis. E.g., could a ratio of theta harmonic power to residual (non theta-harmonic) slow gamma power, or bicoherence measures (e.g., up to the 3rd or 4th harmonic) be useful indicators of the possible errors (by wavelet decomposition). Alternatively, techniques to separate theta harmonics from the remaining (residual) slow gamma band could be suggested.

2) Illustration of methodologies involving experimental data can be further extended. Theta-gamma coupling is a well-used method, and a more extended demonstration of possible issues of this method is useful. In terms of “interaction between gamma frequency and the theta oscillation” (line 429), Figure 7C illustrates a possible problem with the wavelet approach (using scalograms). There was no equivalent presentation of cross-frequency coupling using Fourier decomposition, e.g., using Tort et al. methodology on cross-frequency amplitude-phase coupling (J Neurophysiol 104: 1195). The latter method is based on Fourier decomposition, and it could distinguish sharp (theta harmonics) versus broad coherence peaks in the slow gamma frequency range.

3) In the set of unitary firing data, the authors did not show “phase coupling of neurons to slow gamma versus higher-order theta harmonics in the LFP ”. Coherence spectra (and not power spectra alone; see e.g., Electroencephalogr Clin Neurophysiol 56:668-671) could better study the relation of neuronal firing to the LFPs. Fourier coherence spectrum may show prominent peaks at the theta harmonics (i.e., clearly identifying the theta harmonics).

4) Some sections of the results would benefit from explicit statistical comparisons. The authors largely rely on examples and subjective descriptions of simulated and recorded hippocampal LFPs to support their conclusions. It would be helpful if the authors provided statistical comparisons between the differing analysis methods on the recorded LFP and spike data shown in Figs 6 through 9, and although it is subjectively clear, it would be more convincing if some statistical comparisons were made between the different running speeds and power measures shown in Fig 10.

5) The section, “Comparison of differential recording references”, seems out of place and was not a firmly established point of discussion in the introductory paragraphs. Given that the focus of the manuscript is in the assessment of multiple analytical approaches for decomposing hippocampal LFP, then perhaps this data would be best suited for a complementary methods paper? Alternatively, if the authors wish to retain this data in the current manuscript, perhaps it would be best suited near the end of the results section rather than at the beginning.

6) There are some reservations for some statements made by the authors. These statements relate to conceptual issues of LFP generation or interpretation, which are not critical for this present report.

1. “Any frequency band with non-zero power is, in the Fourier representation, an oscillation”

Comment: This is patently false, since non-oscillatory time series can be represented by a Fourier spectrum as well.

2. “While this synaptic input may be gamma paced in the distal dendrites, within the CA1 pyramidal layer, the frequency is filtered, becoming a bolus of low-frequency (theta) input”

Comment: There are no known models of LFP generation that gamma at the distal dendrites can transform into low-frequency signals. A strongly non-linear process is needed to convert gamma to theta frequency.

3. line 652: straightforward description is that all rhythms in the local field potential are interdependent, coupled by the cascade of energy across spatial and temporal (frequency) scales.

Comment: I am not sure if the present results support interdependence of different rhythms. There is no evidence that cascade of energy across temporal scales has been achieved in the present report. Regarding the ‘cascade’ model, it does not take into consideration the neurobiological evidence of independent gamma frequency oscillation in the basal forebrain and in hippocampal interneurons (gamma resonance in vitro), i.e., gamma frequencies are there without slow frequency driving.

4. Line 516-520: The consequence is that the less coherent activity producing power within the frequency bands surrounding theta and its harmonics is reduced. A parsimonious explanation is that neurons can ‘drift’ (exhibit a range of oscillations)... This accretion of power in one or multiple bands comes at the expense of losing power in others.

Comment: I do not understand this concept of losing power in one frequency while gaining power in another. A pyramidal cell-inhibitory interneuron negative feedback model of gamma generation does describe a decrease in slow gamma accompanied with fast gamma increase.

Minor concerns:

1) Line 179 equation (3): can forward and inverse transform be the same function?

2) In fig. 2, is part (d) (long time period and fine frequency resolution) interchanged with part (h)?

3) Fig. 6f, please label 64 Hz on horizontal axis

4) Line 447: power versus frequency instead?

5) Line 503: which reference with year 2012?

6) Line 573: CA3... region

7) Abstract first line defining LFPs as postsynaptic currents precludes participation of action currents

8) It was unclear from my reading how many sessions of recording LFP data or spike data (from the Buzsaki laboratory) contributed to the analyses shown in each figure.

9) As the authors point out, regional differences in LFP might be an important variable. Thus, it might be useful if the authors speculated on the cortical region serving as a reference in the current study. Do the authors have histological confirmation of recording locations?

10) Fig 6 - it is unclear why the authors included an assessment of “with noise” vs. “no noise”. In addition, on line 21-22, the authors state that the EEMD analysis is not free of harmonic effects. However, the reasons for this statement only allude to the figure. It would be helpful to a general reader if the authors could point out exactly which part of the data in Fig. 6 supports this conclusion.

## Author Response

Synthesis of Reviews:

Synthesis Statement for Author (Required):

This report addresses the possible problem with interpretation of a hippocampal LFP frequency band, the slow gamma of ~20-55 Hz, as an independent rhythm rather than high-order theta harmonics. For artificially generated time signals consisting of only theta and its high-order harmonics, the authors convincingly showed that while short timescale Fourier analysis clearly shows theta harmonics, wavelet and EEMD decomposition display broad power (in spectrograms or scalograms) in the 20-55 Hz range (not clearly identified as harmonics). The authors also provided a clear mathematical reason for such a dispersion, since wavelets of higher order have short time duration and wide frequency dispersion (essentially the Heisenberg uncertainty principle). There can be no dispute that theta harmonics will invade into the slow gamma range, with dispersed peaks. The authors also provided evidence from hippocampal LFPs sorted according to velocity of movement of behaving rats. As theta frequency and power increased with velocity, the power of 20-55 Hz power in the hippocampal LFP actually decreased, unitary 22-55 Hz power of pyramidal cell unitary firing showed no change, while power of interneuronal firing increased in the ~60-100 Hz range. This shows that hippocampal slow gamma varied in a direction opposite to theta harmonics, and that slow gamma responded differently from the theta harmonics.

In our read of this synthesis as well as comments below, we see that some aspects of our initial presentation were unclear. We have made extensive revisions and worked diligently to clarify the text. The suggestion in the synthesis statement “that hippocampal slow gamma varied in a direction opposite to theta harmonics” is an example in which we have now clarified that those data actually refute the presence of slow gamma. Moreover, a reviewer offered that “techniques to separate theta harmonics from the remaining (residual) slow gamma band could be suggested.” Using appropriately tuned parameters, we do not see “slow gamma”. A separation cannot be made. Or alternatively, after Fourier accounts for theta harmonics, there is no slow gamma. We have supported this argument with additional data and analyses that are outlined below.

We openly acknowledge that we are directly challenging the evidence in support of slow gamma by demonstrating that the parameters used by Colgin et al. (2009) in their wavelet analysis (and others later) do not provide a valid measure of the actual time series. Notably, the more recent description of multiple slow gamma bands by Lopes-dos-Santos et al., 2018 suffers from a similar lack of validity for identifying power by frequency. We also openly acknowledge that we cannot speak to all of the existing data. In order to extend our analyses to data from outside our own laboratories, we have included new analyses on data from the Buzsaki lab that are available through the CRCNS database. Specific concerns with the original draft are addressed point by point below.

Major concerns:

1) There is ample evidence that there is a slow gamma frequency band, distinct from the theta harmonics. One piece of evidence is that urethane anesthesia decreased the theta frequency and harmonics, and yet increased slow gamma power (Buzsaki et al., 2003). The authors provided another in terms of slow gamma power decline while theta harmonics increase. For experimental data, while knowing that clear theta harmonics (revealed by power spectra) invade into the slow gamma frequency range, this report does not give an evaluation of how serious this problem could be using wavelet analysis. E.g., could a ratio of theta harmonic power to residual (non theta-harmonic) slow gamma power, or bicoherence measures (e.g., up to the 3rd or 4th harmonic) be useful indicators of the possible errors (by wavelet decomposition). Alternatively, techniques to separate theta harmonics from the remaining (residual) slow gamma band could be suggested.

We will break this into components and address each separately:

“There is ample evidence that there is a slow gamma frequency band, distinct from the theta harmonics.”

The “ample evidence” the reviewer is referring are studies that commit the same error in the application of wavelet. That is, wavelet reliably generates an inaccurate representation of the actual LFP.

This statement is not accurate for three reasons. First, we presented an extensive literature review (figure 1) demonstrating several important points: (1) if a study reported slow gamma, it did not appropriately acknowledge or control for harmonics. (2) if a study acknowledged theta harmonics, the “slow gamma” range was shifted in an attempt to avoid harmonics (e.g., Zheng et al., 2015), or (3) the overlap was acknowledged, but discussion on the overlap was avoided (Cowen et al., 2018). A distinction has never been made between slow gamma and theta harmonics. Thus, to date, there is no available evidence indicating that they could co-exist.

Second, the control for harmonics necessitates the implementation of bicoherence analysis (Aru et al., 2014). To our knowledge, we are the only known group to publish bicoherence analysis with respect to the hippocampal LFP within the past decade.

Third, this comment adopts the position that a distinction can be made. The reviewer assumes that both harmonics and slow gamma “co-exist”. As mentioned above, we cannot find any evidence that this is the case. Wavelet, as implemented by Colgin et al. (2009), shows slow gamma and Fourier shows harmonics. This is not a situation in which a distinction can be made. Rather it is one in which construct validity is challenged (an algorithm fails to measure what it is intended to measure). It is our opinion that the current paper reaffirms the construct validity of Fourier while questioning that of wavelet and EEMD. This is demonstrated through several analyses including the use of a synthetic benchmark dataset with known parameters.

“One piece of evidence is that urethane anesthesia decreased the theta frequency and harmonics, and yet increased slow gamma power (Buzsaki et al., 2003).”

This statement again is both puzzling and inaccurate. First, we do not credit Buzsaki et al. (2003) with the initial report of slow gamma. Rather, the first description occurred 6 years later (Colgin et al., 2009). This reinterpretation is suggestive of confirmation bias, in which there is a tendency to reinterpret data in order to confirm one's existing beliefs.

Second, the citation does not seem to be correct. Pubmed retrieved eight manuscripts from Buzsaki in 2003. None of them fit the description of the reviewer nor describe slow gamma. Furthermore, as Buzsaki did not find it pertinent to carve out a specific band and give it a name, this statement is clearly reinterpreting the past in favor of a specific perspective.

“The authors provided another in terms of slow gamma power decline while theta harmonics increase.”

Previously reported relationships between slow gamma power and velocity is an equivocal topic, as different papers report disparate findings (Kemere et al., 2013 - decrease; Zheng et al., 2015 - little to no change; Chen et al., 2011 - increase). We now address this issue in the manuscript on page 27:

It is worth noting here that the literature is obtuse regarding changes in “slow gamma” with velocity: Zheng and colleagues reported a minimal change with increasing velocity (2015), Kemere et al. reported a decrease (2013), Chen found an increase (2011) and (Ahmed and Mehta, 2012) suggest that there is only one gamma band that increases in frequency and power with velocity. In light of this ambiguity, any potential result risks being construed as supporting “slow gamma”.

Moreover, we apologize for not clearly presenting the main point behind the relationship between velocity and the 25-50 Hz frequency band. We show that as harmonic power increases with velocity, there is a concurrent decrease in the 25-50 Hz frequency band. This power decrease is related to and dependent upon the increase in the harmonics and possibly fast gamma. It is erroneous to suggest that this is ‘slow gamma’ showing a negative correlation with velocity. As written in the manuscript, now on page 28:

...power spectra undergo a redistribution of power across frequencies with velocity.... As described by Ahmed and Mehta (2012), the redistribution of power across frequencies is a consequence of increasing afferent drive onto interneurons (Traub et al., 1996). Specifically, with a low level of afferent input, neurons are weakly entrained exhibiting a range of oscillations. These same neurons become strongly entrained with energy (increasing afferent input) and velocity (Sheremet et al., 2018b). By allowing neurons to interact with each other, a general frequency of entrainment is selected, accompanied by accretion of power in one band with an erosion of power in the adjacent bands (Wiener, 1965, 1966; Strogatz, 1994).

This is further discussed on page 53:

...we observe a spectral reorganization in which some bands increase in power while others decrease as running speed increases (Sheremet et al., 2018a; Sheremet et al., 2019). The drops in power occur between 1-6, 10-14, 19-23, and 25-40 Hz (Figs. 5 and 6). This type of spectral reorganization has been described before, and in fact has a long history. Norbert Wiener observed an increase in the 10 Hz alpha power that was associated with dips (loss of power) in the adjacent frequencies (1965, 1966). The insight was that the global oscillation in the EEG arose from the collective action of the population. As described by Steven Strogatz in his computational model of the phenomenon is: “...that the oscillators interact by pulling on each other's frequencies - if an oscillator is ahead of the group, the group tends to slow it down. If it is going too slowly, the group tends to speed it up. In this way the population of oscillators can achieve a collective enhancement of precision.” (1994, p. 122). Oscillations of the hippocampus do not reside within a single cell or within a pair of cells but across the entire population. As the hippocampus becomes more excitable with running speed, the oscillatory dynamics achieve a “collective enhancement of precision” (see Churchland et al., 2010 for a similar perspective). Under this perspective, the power in 25-50 Hz band at low running speed is a consequence of incohesive oscillatory dynamics among the neurons when not entrained (making the claim that “slow gamma” is a fundamental rhythm dubious; oscillations tend to be equated to entrainment, not incoherence). Once input is large enough, the oscillators fall into an en masse alignment, enhancing the power in some frequency bands at the expense of others.

“For experimental data, while knowing that clear theta harmonics (revealed by power spectra) invade into the slow gamma frequency range, this report does not give an evaluation of how serious this problem could be using wavelet analysis. E.g., could a ratio of theta harmonic power to residual (non theta-harmonic) slow gamma power, or bicoherence measures (e.g., up to the 3rd or 4th harmonic) be useful indicators of the possible errors (by wavelet decomposition). Alternatively, techniques to separate theta harmonics from the remaining (residual) slow gamma band could be suggested.”

Consider this: A person leaves a “haunted house” with photographic evidence of a ghost and takes it to the local college. A researcher looks at the camera and the photo and, by knowing how that camera works, understands how its tendency for over-exposure may give the impression of a ghost (an invalid measure of the environment). They demonstrate this by 1) replicating the artifact, generating the false image of a ghost and 2) documenting that with other cameras and scientific equipment, one cannot resolve the image of a specter (using a valid approach). In this instance, there is no technique that can separate “ghost” from “non-ghost”.

The same is true with respect to “slow gamma” versus harmonics. Wavelet decomposition as done by Colgin et al. (2009) results in a reliable, yet invalid representation of the time-series. In the revised manuscript we now show different parameters of wavelet that also converge on harmonics. In light of this, which version of wavelet are we to believe? The one that maintains validity with the synthetic benchmark timeseries (see Figure 11).

Given the available evidence from the direct comparison of wavelet and Fourier, “...neither of these two decompositions offer a “mixed representation” where both harmonics and slow gamma are present” (This statement now appears in the manuscript on page 44). There is no distinction to be made.

The reviewer asks: How serious is the problem? Effectively, so serious that we challenge both the initial description of “slow gamma” using wavelet and the more recent EEMD decomposition on the grounds of using invalid decomposition measures. We have addressed this potential crisis in several new analysis in the revised version:

Fig. 5- using data from the Buzsaki laboratory and publicly available for download, we demonstrate that there is neither a peak above noise in the “slow gamma” range nor cross-frequency coupling that supports “slow gamma”. We implemented bicoherence here per the reviewer's suggestion.

Fig. 7- also using data from the Buzsaki laboratory we emphasize two important points. First, finding modulation in a frequency band is not evidence of a fundamental rhtyhm as there is spike frequency modulation across all frequencies. Second, neurons are first and foremost modulated by theta (there are no neruons that solely prefer “slow gamma”/“fast gamma”). Third, there is no evidence that spikes are modulated by “slow gamma”.

Fig. 8 - Power spectral density of spike trains do not show “slow gamma” modulation. Spike-LFP coherence is incredibly low to the point that it could be considered “incoherent” (see Buzsaki and Schomburg, 2015 for the interpretation with respect to low coherence values). Nevertheless, peaks in coherence are seen at theta, 14-18 Hz harmonic and the 21-27 Hz harmonic.

Fig. 10 - EEMD fails to account for harmonics, questioning the claims and validity of the Lopes-dos-Santos et al. (2018) results with respect to “slow gamma” bands.

Fig. 11 - Different wavelet parameters converge on different answers! Using a wavelet of higher time support, the harmonics present in the synthetic trace are resolved. The parameters of Colgin et al. (2009) converge on an invalid “slow gamma”.

Fig. 12- “Slow gamma” epochs have been previously found by finding instances of time in which the power exceeds 2 standard deviations above the mean. We used this method to identify instances of both ripples (highly transient events) as well as “slow gamma” events. When triggered, Fourier decomposition is capable of resolving ripple power. When the same procedure is applied to the 25-50 Hz frequency band, Fourier resolves harmonics of theta. Calculating wavelet on these segments shows a broad “slow gamma” bump. Again, both cannot be correct. In accord with the reviewer's suggestion, we ran bicoherence finding only theta harmonics.

In addressing the statement “...this report does not give an evaluation of how serious this problem could be using wavelet analysis” - While there is no metric for ‘seriousness’ of using invalid analytical tools, we feel that it is important to return to basic principles of spectral decomposition and engage in discussions of “construct validity”. The “ample evidence for slow gamma” that the reviewer refers to is set upon the foundation of an inappropriately tuned Morlet wavelet. That being said, we prefer to leave it to the reader to gauge the seriousness of the issue.

2) Illustration of methodologies involving experimental data can be further extended. Theta-gamma coupling is a well-used method, and a more extended demonstration of possible issues of this method is useful. In terms of “interaction between gamma frequency and the theta oscillation” (line 429), Figure 7C illustrates a possible problem with the wavelet approach (using scalograms). There was no equivalent presentation of cross-frequency coupling using Fourier decomposition, e.g., using Tort et al. methodology on cross-frequency amplitude-phase coupling (J Neurophysiol 104: 1195). The latter method is based on Fourier decomposition, and it could distinguish sharp (theta harmonics) versus broad coherence peaks in the slow gamma frequency range.

We have now included two additional instances of higher-order spectral decomposition in figures 5 and 12. Please also note that we presented the Fourier alternative in a prior manuscript (Sheremet et al., 2019).

First, regarding the scalogram, we do not wish to give the impression that this scalogram is absolute. Therefore, we present scalograms with different parameters, two that do not maintain fidelity with the synthetic trace (invalid; wavelet ratio is too small) and one that can resolve the harmonics (valid and aligned with the results of Fourier; see Fig. 11).

The purpose of the scalogram presentation in our manuscript is to demonstrate how the variable time-frequency representation inherent to wavelet has the potential to convolve the harmonics. Specifically, to achieve precision within a theta cycle (high temporal precision), Colgin et al. (2009) sacrificed frequency resolution at the cost of bleeding over harmonics. We again point to the definition of “construct validity”, that is, “the degree to which a test measures what it claims, or purports, to be measuring” (per Wikipedia).

The intention of this figure is to demonstrate that Colgin's wavelet does not maintain construct validity. To enhance on this presentation, we demonstrate parameters of wavelet decomposition that are capable of converging on a more valid representation. The concern resides in the inability of the method to measure what it is intended to measure.

Regarding the Fourier alternative, we provided an extensive methodological description demonstrating that a time-frequency trade-off exists in any decomposition method (see Figures 2 and 4). The reviewer appears to request a similar scalogram based on Fourier decomposition. As described, should the time support for Fourier be too narrow, the frequency resolution is destroyed. Simply, trimming down Fourier to temporal windows that are less than a theta cycle risks the same mistake as Colgin's wavelet resulting in an erroneous presentation.

The reviewer suggests that the Tort manuscript as a method to circumvent this issue. In their manuscript, Tort and colleagues looked at the coupling between a wideband (e.g, 60-100 Hz) and theta. What does not seem to be appreciated by this approach is that it is effectively a “poor man's bispectral analysis” (Kovach et al., 2018; PMID: 29481965). We can illustrate this using the animation available here (https://en.wikipedia.org/wiki/Fourier_series#/media/File:Fourier_series_and_transform.gif)

Consider this square wave:

[Redacted figure was a picture of a square wave]

Fourier will decompose it into a sum of sine waves:

[Redacted figure was a picture of the sum of sine wave oscillations that would combine to a square wave]

And each sinusoid in the Fourier decomposition is of a fixed amplitude:

[Redacted figure was a picture showing that each specific sine wave of a certain frequency is fixed in amplitude]

power spectra (one amplitude per freq.)

Based on the way that Fourier works, when decomposed into the sum of sine waves, a specific frequency has a fixed amplitude. One cannot run a Fourier decomposition and find a varying amplitude in a narrow frequency band. That is, in one realization, the amplitude of a 70 Hz oscillation is fixed- no amplitude modulation.

So how did Tort get their results of modulation in a Fourier based method? They opened up the band to be between 30-60 or 60-100 Hz:

[Redacted figure was a picture adding together two sine waves of a slightly different frequencies - see https://en.wikipedia.org/wiki/Beat_(acoustics) for an illustration]

By keeping the frequency range wide, Tort et al. permitted an “oscillatory beating,” which gives the impression of amplitude modulation. The upper frequency limit of the “oscillatory beating” is bounded by the bandwidth of the filter.

Figure from (https://slideplayer.com/slide/4586601/).

Simply stated, the amplitude modulation reported by Tort can be interpreted as two oscillations interacting. To ask the question “how does this beating amplitude relate to the phase of theta?” becomes a 3-oscillation interaction.

This analysis is known as the bispectrum (see figure below from Kovach et al., 2018). Kovach discusses the ins-and-outs of bispectral decomposition relative to other methods (including Tort et al., 2010). In this manuscript, he demonstrates that “traditional measures of phase-amplitude coupling tend to fall short when compared to bispectral analysis (Kovach et al., 2018)”. This statement now appears in our manuscript.

[Redacted images was Fig. 1 from Kovach et al., 2018]

Now one may say “That's great! Why don't you do that analysis?”. We have done this analysis and published it previously (along with statistical comparisons). Please see figures 8 and 9 of Sheremet et al., 2019. Notably, the data presented in Sheremet et al., (2019) are consistent with the analyses and interpretation presented in the current paper.

3) In the set of unitary firing data, the authors did not show “phase coupling of neurons to slow gamma versus higher-order theta harmonics in the LFP ”. Coherence spectra (and not power spectra alone; see e.g., Electroencephalogr Clin Neurophysiol 56:668-671) could better study the relation of neuronal firing to the LFPs. Fourier coherence spectrum may show prominent peaks at the theta harmonics (i.e., clearly identifying the theta harmonics).

We thank the reviewer for this insightful suggestion and have added these analyses. Specifically, we have now conducted Spike-LFP analysis showing harmonic interactions as well as replicated the “depth of modulation/phase preference” of Schomburg et al. (2014). Please see new figures 7 and 8.

4) Some sections of the results would benefit from explicit statistical comparisons. The authors largely rely on examples and subjective descriptions of simulated and recorded hippocampal LFPs to support their conclusions. It would be helpful if the authors provided statistical comparisons between the differing analysis methods on the recorded LFP and spike data shown in Figs 6 through 9, and although it is subjectively clear, it would be more convincing if some statistical comparisons were made between the different running speeds and power measures shown in Fig 10.

Statistical comparisons are generally applied when comparing two or more populations, or samples from presumed different populations. In this manuscript, we are not comparing samples or populations.

Perhaps it is being implied that we statistically compared decompositions? This would be a strange thing to do. When given a synthetic series, a single algorithm (either wavelet and Fourier) will always converge to the same answer. This is not a stochastic process and there is no variance across iterations of the same algorithm. Furthermore, running a statistical comparison on different methods on LFP is opaque as we now show that two different Morlet wavelets give contrary representations (Figure 11; wavelet ratio of 7 shows slow gamma, 30 shows harmonics). Running a “statistical comparison” within wavelet seems even more obtuse. The difference across wavelet decomposition can be directly accounted for by the selected parameters. As mathematical decompositions involve the “plug and chug” of numbers, there is no surprise that there is a reliable difference between each wavelet decomposition when we use different mother wavelets. Statistical comparison here, therefore is not meaningful.

It also loses the actual theme of the manuscript. We are not challenging the approaches in terms of reliability, but rather providing evidence that there is insufficient validity.

For instance, there are multiple ways to assess gene expression - Northern blot, microarrays, RT-PCR and inferring expression through protein level (Western blot). Each method has its own characteristics with advantages and drawbacks with respect to the question being asked. But are they statistically different approaches? If two approaches had (1) consistent but (2) contradictory results, is this difference statistically significant? That's an odd question comparing apples to oranges.

With respect to the suggestion that we statistically assess power as a function of velocity... we did that in Sheremet et al., (2019). From the abstract:

“Across layers, we found a reliable correlation between the power of theta and the power of gamma, indicative of an amplitude-amplitude relationship. Moreover, there was an increase in the coherence between the power of gamma and the phase of theta, demonstrating increased phase-amplitude coupling with speed. Finally, at higher velocities, phase entrainment between theta and gamma increases.”

Note that the issue of statistical comparison, neither enhances nor diminishes the importance of the current manuscript - Colgin's wavelet, which has been replicated across multiple studies, does not provide a valid representation of hippocampal LFP.

5) The section, “Comparison of differential recording references”, seems out of place and was not a firmly established point of discussion in the introductory paragraphs. Given that the focus of the manuscript is in the assessment of multiple analytical approaches for decomposing hippocampal LFP, then perhaps this data would be best suited for a complementary methods paper? Alternatively, if the authors wish to retain this data in the current manuscript, perhaps it would be best suited near the end of the results section rather than at the beginning.

We agree and this section has now been removed.

6) There are some reservations for some statements made by the authors. These statements relate to conceptual issues of LFP generation or interpretation, which are not critical for this present report.

1. “Any frequency band with non-zero power is, in the Fourier representation, an oscillation”

Comment: This is patently false, since non-oscillatory time series can be represented by a Fourier spectrum as well.

This appears to be a misunderstanding, and we apologize for not using clearer language in the previous draft.

We have now rephrased the statement to read “Fourier and wavelet decompositions both yield representations in which there is power across all frequencies (Fig. 9).”

2. “While this synaptic input may be gamma paced in the distal dendrites, within the CA1 pyramidal layer, the frequency is filtered, becoming a bolus of low-frequency (theta) input”

Comment: There are no known models of LFP generation that gamma at the distal dendrites can transform into low-frequency signals. A strongly non-linear process is needed to convert gamma to theta frequency.

We apology for the lack of clarity. The manuscript has been reworked and this sentiment is now described as:

However, dendrites operate as low pass filters (Golding et al., 2005) in which synaptic input may be gamma paced at the dendrites, but become a bolus of low-frequency (theta) input at the soma (Vaidya and Johnston, 2013).

Please note that others have written that dendrites operate as the non-linear processors in this instance. The citations we have now included capture this point:

• Vaidya SP, Johnston D (2013) Temporal synchrony and gamma-to-theta power conversion in the dendrites of CA1 pyramidal neurons. Nat Neurosci 16:1812-1820.

• Golding NL, Mickus TJ, Katz Y, Kath WL, Spruston N (2005) Factors mediating powerful voltage attenuation along CA1 pyramidal neuron dendrites. J Physiol 568:69-82.

3. line 652: straightforward description is that all rhythms in the local field potential are interdependent, coupled by the cascade of energy across spatial and temporal (frequency) scales.

Comment: I am not sure if the present results support interdependence of different rhythms. There is no evidence that cascade of energy across temporal scales has been achieved in the present report. Regarding the ‘cascade’ model, it does not take into consideration the neurobiological evidence of independent gamma frequency oscillation in the basal forebrain and in hippocampal interneurons (gamma resonance in vitro), i.e., gamma frequencies are there without slow frequency driving.

It would be a rare instance in which a single publication is capable of supplanting one theory for another. That is, verification of the Cascade hypothesis is certainly not a matter to be settled in a single manuscript focusing on spectral decomposition methods. Rather, it has been the topic of our prior two publications (Sheremet et al., 2018; Sheremet et al., 2019) and an idea that we will continue to rigorously test as we move forward. However, a major obstacle in making headway for the cascade theory is the notion of “multiplexed oscillations.”

Multiplexing was born out of the observation of “two independent gamma rhythms.” Can both multiplexing and cascade be correct? Perhaps. However, the current data challenge the idea of spectral multiplexing as it is evident that the data that launched the theory is not a valid representation of the biology. We feel that it is pertinent to discuss an alternative to the multiplexing hypothesis, which is the energy cascade model.

We look forward to engaging in this dialog further as our research progresses, but will emphasize that in Gyuri Buzsaki's book “Rhythms of the Brain”, he defends the cascade perspective:

“Indeed, the inverse relationship between frequency and its power is an indication that there is a temporal relationship between frequencies: perturbations of slow frequencies cause a cascade of energy dissipation at all frequency scales. One may speculate that these interference dynamics are the essence of the global temporal organization of the cortex.” (p.121)

The reviewer adopts the argument that there is a gamma in the absence of slow frequency driving. This is not accurate in our opinion. In any power spectra, the highest power is in the lowest frequency (see p. 121 of Buzsaki, 2006). That is, the power in the 2Hz band would eclipse the power in the 60 Hz band by at least one order of magnitude. Therefore, there is always the potential for a slow frequency drive for gamma. It needs to be examined before being discounted.

4. Line 516-520: The consequence is that the less coherent activity producing power within the frequency bands surrounding theta and its harmonics is reduced. A parsimonious explanation is that neurons can “drift” (exhibit a range of oscillations)... This accretion of power in one or multiple bands comes at the expense of losing power in others.

Comment: I do not understand this concept of losing power in one frequency while gaining power in another. A pyramidal cell-inhibitory interneuron negative feedback model of gamma generation does describe a decrease in slow gamma accompanied with fast gamma increase.

We appreciate that this sentiment may lack clarity. We have now rewritten this section and included citations:

...The drops in power occur between 1-6, 10-14, 19-23, and 25-40 Hz (Figs. 5 and 6). This type of spectral reorganization has been described before, and in fact has a long history. Norbert Wiener observed an increase in the 10 Hz alpha power that was associated with dips (loss of power) in the adjacent frequencies (1965, 1966). The insight was that the global oscillation in the EEG arose from the collective action of the population. As described by Steven Strogatz in his computational model of the phenomenon is: “...that the oscillators interact by pulling on each other's frequencies - if an oscillator is ahead of the group, the group tends to slow it down. If it is going too slowly, the group tends to speed it up. In this way the population of oscillators can achieve a collective enhancement of precision.” (1994, p. 122). Oscillations of the hippocampus do not reside within a single cell or within a pair of cells but across the entire population...

Thus, from the Strogatz perspective, “slow gamma” is actually incoherent activity.

Here is a figure from Steven Strogatz (1994):

[Redacted figure was figure 1 from Strogatz, 1994 including the quote from Nortber Wiener]

Minor concerns:

1) Line 179 equation (3): can forward and inverse transform be the same function?

The function is g and the transform is G. While we understand that they may look similar, they are not in principle the same function. Close inspection will reveal one is the integral and the other is the double integral.

We refer the reviewer to Daubechies, Ingrid. Ten lectures on wavelets. Vol. 61. Siam, 1992.

2) In fig. 2, is part (d) (long time period and fine frequency resolution) interchanged with part (h)?

Thank you. This has been corrected.

3) Fig. 6f, please label 64 Hz on horizontal axis

We have now labeled the entirety of the range.

4) Line 447: power versus frequency instead?

Corrected.

5) Line 503: which reference with year 2012?

Apologies. Ahmed and Mehta (2012). This correction has been made.

6) Line 573: CA3... region

Corrected.

7) Abstract first line defining LFPs as postsynaptic currents precludes participation of action currents

This line now reads “Local field potential (LFP) oscillations are primarily shaped by the superposition of postsynaptic currents.”

8) It was unclear from my reading how many sessions of recording LFP data or spike data (from the Buzsaki laboratory) contributed to the analyses shown in each figure.

Apologies. Our methods now read:

“Single-unit data were generously provided by the Buzsaki laboratory and curated by the Collaborative Research in Computational Neuroscience (Mizuseki et al., 2014; Pastalkova et al., 2015; 7 datasets: maze05.005, maze06.002, i01_maze06.005, i01_maze08.001, i01_maze08.004, maze13.003, and maze15.002).”

9) As the authors point out, regional differences in LFP might be an important variable. Thus, it might be useful if the authors speculated on the cortical region serving as a reference in the current study. Do the authors have histological confirmation of recording locations?

We do have histological verification should it be warranted. But it should suffice to say that, as our manuscript is comparing the validity of different methods to time-series analysis, anatomical verification would neither enhance nor detract from our presentation. The same time-series (synthetic and real) we processed through the algorithms. Differences are accounted for by the method, not electrode location. Furthermore, the section on references has now been removed. Since arriving at the University of Florida, we never used a cortical reference.

We have described that we placed “...a reference over the cerebellum” in the methods section.

The reference electrode location is the same for the new data included from the Buzsaki laboratory (also cerebellar reference).

In order to localize electrode location, we implemented current source density in both our own data and the Buzsaki data (see figure 5; note how this method resolve the hippocampal anatomy quite well). We also stated that in the methods that, with our linear electrode arrays, “...the position of the hippocampal layers with respect to the recording channels was determined by estimating the distribution of current-source density to identify the CA1 pyramidal cell layer (Rappelsberger et al., 1981; Mitzdorf, 1985; Buzsaki et al., 1986; Bragin et al., 1995).” Should the reviewer wish to examine our placement, we offer the figure published in our last manuscript examining theta-gamma coupling across layers (Sheremet et al., 2019).

10) Fig 6 - it is unclear why the authors included an assessment of “with noise” vs. “no noise”. In addition, on line 21-22, the authors state that the EEMD analysis is not free of harmonic effects. However, the reasons for this statement only allude to the figure. It would be helpful to a general reader if the authors could point out exactly which part of the data in Fig. 6 supports this conclusion.

Apologies for leaving this out. We have now stated in the caption of this figure (Now Fig 9): “The inclusion of pink noise provides a more realistic spectrum for comparison to the raw LFP.”

The reviewer brings up a great point. We have now introduced a bicoherence analysis of the EEMD output which shows that post-EEMD still contains harmonics (Fig 10).

-

Aru J, Priesemann V, Wibral M, Lana L, Pipa G, Singer W, Vicente R (2014) Untangling cross-frequency coupling in neuroscience. Curr Opin Neurobiol 31C:51-61.

Berényi A, Somogyvári Z, Nagy AJ, Roux L, Long JD, Fujisawa S, Stark E, Leonardo A, Harris TD, Buzsáki G (2014) Large-scale, high-density (up to 512 channels) recording of local circuits in behaving animals. J Neurophysiol 111:1132-1149.

Bieri KW, Bobbitt KN, Colgin LL (2014) Slow and fast γ rhythms coordinate different spatial coding modes in hippocampal place cells. Neuron 82:670-681.

Buzsáki G, Draguhn A (2004) Neuronal oscillations in cortical networks. Science 304:1926-1929.

Golding NL, Mickus TJ, Katz Y, Kath WL, Spruston N (2005) Factors mediating powerful voltage attenuation along CA1 pyramidal neuron dendrites. J Physiol 568:69-82.

Kamondi A, Acsády L, Wang XJ, Buzsáki G (1998) Theta oscillations in somata and dendrites of hippocampal pyramidal cells in vivo: activity-dependent phase-precession of action potentials. Hippocampus 8:244-261.

Maurer AP, Burke SN, Lipa P, Skaggs WE, Barnes CA (2012) Greater running speeds result in altered hippocampal phase sequence dynamics. Hippocampus 22:737-747.

McNaughton BL, Barnes CA, O'Keefe J (1983) The contributions of position, direction, and velocity to single unit activity in the hippocampus of freely-moving rats. Exp Brain Res 52:41-49.

Mizuseki K, Diba K, Pastalkova E, Teeters J, Sirota A, Buzsáki G (2014) Neurosharing: large-scale data sets (spike, LFP) recorded from the hippocampal-entorhinal system in behaving rats. F1000Res 3:98.

Pastalkova E, Wang Y, Mizuseki K, Buzsáki G (2015) Simultaneous extracellular recordings from left and right hippocampal areas CA1 and right entorhinal cortex from a rat performing a left / right alternation task and other behaviors. . In. http://dx.doi.org/10.6080/K0KS6PHF: CRCNS.org.

Quilichini P, Sirota A, Buzsáki G (2010) Intrinsic circuit organization and theta-gamma oscillation dynamics in the entorhinal cortex of the rat. J Neurosci 30:11128-11142.

Rappelsberger P, Pockberger H, Petsche H (1981) Current source density analysis: methods and application to simultaneously recorded field potentials of the rabbit's visual cortex. Pflugers Arch 389:159-170.

Sheremet A, Burke SN, Maurer AP (2016) Movement Enhances the Nonlinearity of Hippocampal Theta. J Neurosci 36:4218-4230.

Sheremet A, Qin Y, Kennedy JP, Zhou Y, Maurer AP (2018) Wave turbulence and energy cascade in the hippocampus. Frontiers in Systems Neuroscience 12:62.

Sheremet A, Kennedy JP, Qin Y, Zhou Y, Lovett SD, Burke SN, Maurer AP (2019) Theta-gamma cascades and running speed. J Neurophysiol 121:444-458.

Skaggs WE, McNaughton BL, Wilson MA, Barnes CA (1996) Theta phase precession in hippocampal neuronal populations and the compression of temporal sequences. Hippocampus 6:149-172.

Strogatz SH (1994) Norbert Wiener's brain waves. In: Frontiers in Mathematical Biology, pp 122-138: Springer.

Vaidya SP, Johnston D (2013) Temporal synchrony and gamma-to-theta power conversion in the dendrites of CA1 pyramidal neurons. Nat Neurosci 16:1812-1820.

Wiener N (1965) Cybernetics or Control and Communication in the Animal and the Machine: MIT press.

Wiener N (1966) Nonlinear problems in random theory. Nonlinear Problems in Random Theory, by Norbert Wiener, pp 142 ISBN 0-262-73012-X Cambridge, Massachusetts, USA: The MIT Press, August 1966(Paper):142.

Yamaguchi Y, Aota Y, McNaughton BL, Lipa P (2002) Bimodality of theta phase precession in hippocampal place cells in freely running rats. J Neurophysiol 87:2629-2642.

Zheng C, Bieri KW, Hsiao YT, Colgin LL (2016) Spatial Sequence Coding Differs during Slow and Fast Gamma Rhythms in the Hippocampus. Neuron 89:398-408.
